# Organization and architecture of AggR‐dependent promoters from enteroaggregative *Escherichia coli*

**DOI:** 10.1111/mmi.14172

**Published:** 2018-12-18

**Authors:** Muhammad Yasir, Christopher Icke, Radwa Abdelwahab, James R. Haycocks, Rita E. Godfrey, Pavelas Sazinas, Mark J. Pallen, Ian R. Henderson, Stephen J. W. Busby, Douglas F. Browning

**Affiliations:** ^1^ Institute of Microbiology and Infection, School of Biosciences University of Birmingham Birmingham B15 2TT UK; ^2^ Quadram Institute Bioscience Norwich Research Park Norwich NR4 7UA UK; ^3^ Faculty of Medicine Assiut University Assiut Egypt; ^4^ Department of Biotechnology and Biomedicine Technical University of Denmark DK‐2800 Kgs Lyngby Denmark

## Abstract

Enteroaggregative *Escherichia coli* (EAEC), is a diarrhoeagenic human pathogen commonly isolated from patients in both developing and industrialized countries. Pathogenic EAEC strains possess many virulence determinants, which are thought to be involved in causing disease, though, the exact mechanism by which EAEC causes diarrhoea is unclear. Typical EAEC strains possess the transcriptional regulator, AggR, which controls the expression of many virulence determinants, including the attachment adherence fimbriae (AAF) that are necessary for adherence to human gut epithelial cells. Here, using RNA‐sequencing, we have investigated the AggR regulon from EAEC strain 042 and show that AggR regulates the transcription of genes on both the bacterial chromosome and the large virulence plasmid, pAA2. Due to the importance of fimbriae, we focused on the two AAF/II fimbrial gene clusters in EAEC 042 (*afaB‐aafCB* and *aafDA*) and identified the promoter elements and AggR‐binding sites required for fimbrial expression. In addition, we examined the organization of the fimbrial operon promoters from other important EAEC strains to understand the rules of AggR‐dependent activation. Finally, we generated a series of semi‐synthetic promoters to define the minimal sequence required for AggR‐mediated activation and show that the correct positioning of a single AggR‐binding site is sufficient to confer AggR‐dependence.

## Introduction

Enteroaggregative *Escherichia coli* (EAEC) is an important human pathogen that is responsible for causing diarrhoea in both adults and children in industrialized and developing countries (Okeke *et al.*, 2000; Wilson *et al.*, 2001; Nataro *et al.*, 2006; Franca *et al.*, 2013). It has been shown to elicit travellers’ diarrhoea, paediatric diarrhoea and persistent diarrhoea in HIV‐infected patients, as well as extra‐intestinal infections, such as urinary tract infections and septicaemia (Durrer *et al.*, 2000; Okeke *et al.*, 2000; Adachi *et al.*, 2001; Olesen *et al.*, 2012; Herzog *et al.*, 2014). EAEC strains have been linked to a number of serious diarrhoeal outbreaks, including the food‐borne outbreak caused by a Shiga‐toxin‐producing EAEC O104:H7 in Germany in 2011, which infected over 4000 individuals and resulted in 54 deaths (Itoh *et al.*, 1997; Harada *et al.*, 2007; Frank *et al.*, 2011; Boisen *et al.*, 2015). In spite of its global importance as a human pathogen, the mechanisms by which EAEC causes disease are still poorly understood. In some instances, specific virulence determinants have been identified, but as EAEC strains are extremely heterogeneous in nature, many determinants are not present in all strains (Estrada‐Garcia and Navarro‐Garcia, 2012; Franca *et al.*, 2013).

EAEC pathogenesis is thought to proceed by the colonization of the human intestinal mucosa followed by the production of various toxins, such as plasmid‐encoded toxin (Pet), the Pic mucinase, enteroaggregative heat‐stable toxin (EAST‐1) and *Shigella *enterotoxin 1 (ShET1), and the concurrent triggering of inflammation (Savarino *et al.*, 1991; Fasano *et al.*, 1997; Henderson *et al.*, 1999; Harrington *et al.*, 2009; Estrada‐Garcia and Navarro‐Garcia, 2012). Typical EAEC strains carry the plasmid‐encoded AggR transcription regulator protein, a member of the AraC‐XylS family of transcription factors (Nataro *et al.*, 1994; Sarantuya *et al.*, 2004). AggR co‐ordinately activates the expression of many genes thought to be required for pathogenesis, for example the attachment adherence fimbriae (AAF) required for colonization, the anti‐aggregative protein dispersin (Aap) and its dedicated type I secretion system (T1SS) (Elias *et al.*, 1999; Sheikh *et al.*, 2002; Nishi *et al.*, 2003; Morin *et al.*, 2013). As AggR is central to activating the expression of essential virulence genes, it is key to understanding pathogenesis in this important human pathogen. Here, we use RNA‐sequencing (RNA‐seq) to examine the AggR regulon in the pathogenic EAEC strain 042 and show that AggR regulates genes on both the large virulence plasmid, pAA2, and the bacterial chromosome. As fimbrial biogenesis is central to EAEC pathogenesis, we examine the organization and architecture of AggR‐dependent fimbrial promoters from EAEC strain 042 and from a number of other important EAEC strains, identifying the different promoter elements and functional AggR‐binding sites required for expression.

## Results

### RNA‐seq analysis of the AggR regulon in EAEC strain 042

**Figure 1:**
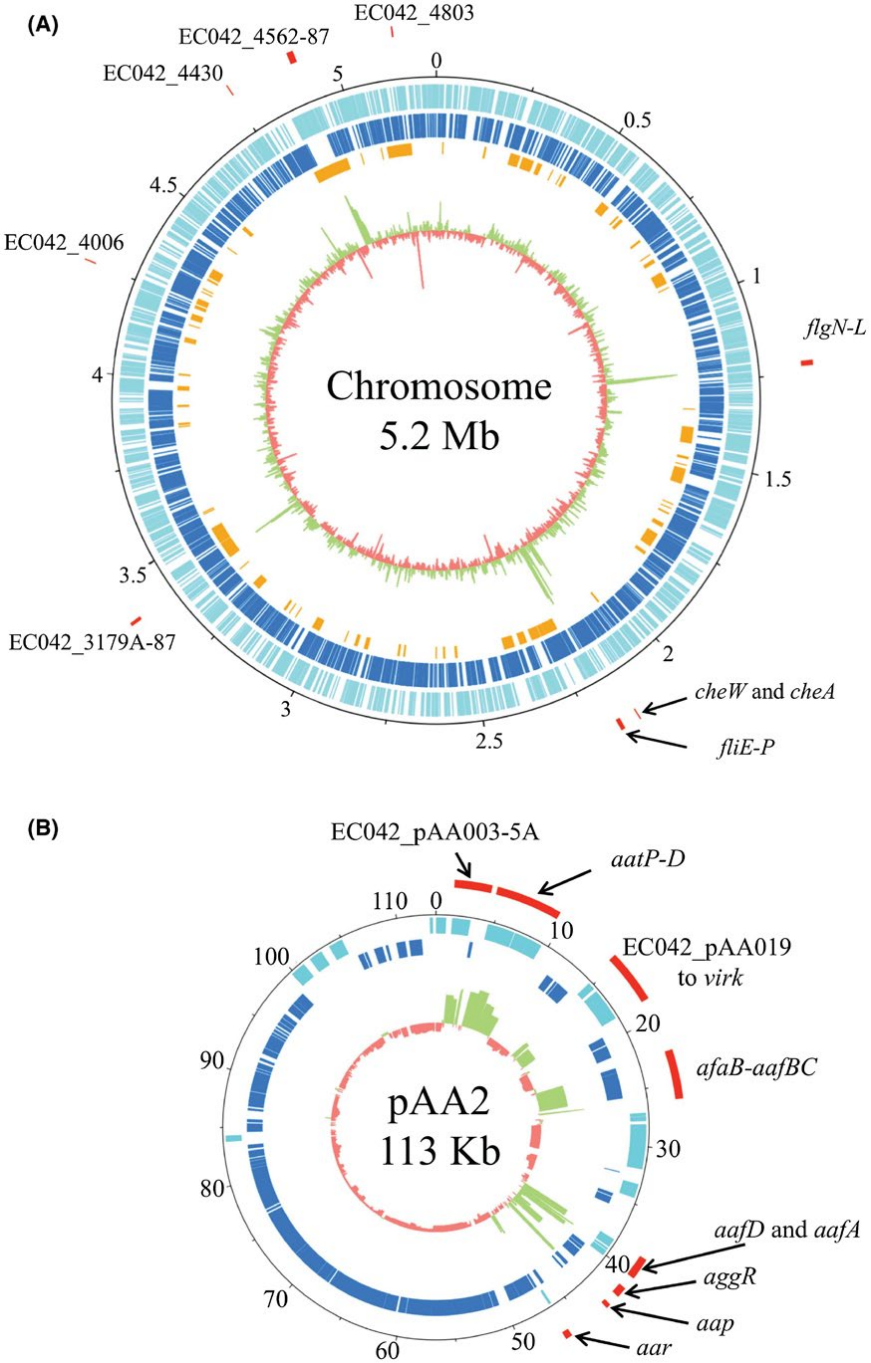
AggR‐regulated genes in EAEC strain 042. The figure shows the differential gene expression observed between wild‐type EAEC 042 and its *aggR *mutant on A. the chromosome and B. plasmid pAA2, as determined by RNA‐seq. A. The data are displayed in rings from the outside inwards. The outermost red lines identify some of the differentially expressed genes (which are labelled with their gene name or number), followed by the base coordinates of the chromosome (labelled in Mb). The annotated genes of EAEC 042 are indicated in the forward and reverse orientation (light blue and dark blue respectively). The EAEC 042 chromosomal regions of difference (RODs) as identified by Chaudhuri *et al.* (2010) are presented in orange. The inner most circle shows the log_2_ fold difference for each gene compared between wild‐type EAEC 042 and the *aggR *mutant. Positively differential expressed genes are presented in green and negatively differentially expressed genes are in red. B. The rings depicting the data for plasmid pAA2 are the same as for the EAEC 042 chromosome in A. Note that base numbering for pAA2 is in Kb.

AggR is the master regulator of EAEC virulence. Previously, Morin *et al. *(2013) examined the AggR regulon, using micro‐arrays, for the archetypal pathogenic strain EAEC 042. As micro‐array analysis can be influenced by probe design and genome annotation, and has issues with detecting low abundance transcripts (Zhao *et al.*, 2014), we repeated the analysis using high‐throughput RNA‐seq methodology. Briefly, wild‐type EAEC 042 and an isogenic *aggR *mutant strain (EAEC 042 Δ*aggR*) (Table S1) were grown in triplicate until mid‐logarithmic growth in high glucose Dulbecco’s modified Eagle’s medium (DMEM), which has been shown to induce biofilm formation and AggR‐dependent gene expression in EAEC (Sheikh *et al.*, 2001; Morin *et al.*, 2013), and RNA was isolated and contaminating DNA removed. The isolated RNA was converted to cDNA and sequenced, generating over 7 million reads each with > 90% of reads aligning to the EAEC 042 genome. Genes were considered to be differentially expressed if there was > 1 log_2_‐fold difference in expression accompanied by an adjusted *p*‐value < 0.00001 between the mutant and the wild‐type strains. In total, 112 genes were differentially expressed in EAEC 042 in comparison to the *aggR *mutant (Tables S2 and S3). These genes were located on both the chromosome and the large pAA2 plasmid (Fig. 1). Note that with the exception of EC042_pAA056, all the AggR‐regulated genes identified by Morin *et al. *(2013) were identified by our study (Tables S2 and S3).

Of the 112 genes that showed differential expression between the wild‐type and the *aggR *mutant, 29 were located in clusters on the large virulence plasmid, pAA2 (Fig. 1B and Table S2). It is of note that these genes are all confined to one half of the plasmid, whilst the genes required for plasmid replication and conjugative transfer are located on the other half and are independent of AggR control (Fig. 1B) (Chaudhuri *et al.*, 2010). The expression of many of these genes has previously been shown to be dependent on AggR, for example *aggR* itself, *aar*, which encodes a repressor of AggR, the five genes encoding the Aat T1SS (*aatPABCD*) and its secreted substrate dispersin (*aap*), EC042_pAA003 and EC042_pAA004 that encode proteins that are involved in biofilm formation, the polysaccharide deacetylase encoded by *shf*, the *Shigella flexneri* virulence protein VirK and the AAF/II fimbriae (*aafDA *and *afaB‐aafCB*) (Elias *et al.*, 1999; Nishi *et al.*, 2003; Fujiyama *et al.*, 2008; Chaudhuri *et al.*, 2010; Morin *et al.*, 2010; Morin *et al.*, 2013; Santiago *et al.*, 2014). Many of the genes, which are encoded on pAA2 and activated by AggR, have unknown function (*e.g. *EC042_pAA005, EC042_pAA005A, EC042_pAA019, EC042_pAA020 and EC042_pAA061) and, thus, their potential role in EAEC 042 pathogenicity is unclear.

From the genes differentially expressed in the *aggR *mutant, 83 were located on the chromosome and many of these genes are located in chromosomal islands (Fig. 1A and Table S3), for example the genes which encode the Aai type VI secretion system (T6SS) (EC042_4562 to EC042_4583 (*aaiA *to* aaiU*)). This cluster consists of the 16 genes encoding the T6SS machinery and 4 hypothetical proteins (EC042_4580, EC042_4581, EC042_4582 and EC042_4583) and has been shown to be activated by AggR (Dudley *et al.*, 2006; Morin *et al.*, 2013). A second AggR activated chromosomal island extends from EC042_3179A to EC042_3187 (Fig. 1A and Table S3). Previously, Morin *et al. *(2013) identified EC042_3182 and EC042_3184 as being AggR regulated. Whilst many genes within this region encode conserved proteins, with no homology to any known protein family (*e.g. *EC042_3179A, EC042_3180 and EC042_3184), EC042_3181 is of note as it is homologous to the transcription activator PerC from enteropathogenic *E. coli*, which regulates the *LEE1 *pathogenicity island (Knutton *et al.*, 1997).

Interestingly, genes associated with flagellar motility were down regulated in the *aggR *mutant, whilst antigen 43 (Agn43) homologues (EC042_4803, *flu1*, and *flu2*) were up regulated, suggesting that AggR might regulate motility and cell aggregation in EAEC 042 (Table S3). To investigate this, the relative expression of three flagella genes (*fliA*, *flgB* and *fliC*) and EC042_4803 was assessed by qRT‐PCR. Results in Fig. S1 and Table S4 demonstrated that there was no statistically significant difference in the expression of *fliA*, *flgB*, *fliC *or EC042_4803 between the wild‐type EAEC 042 and the *aggR* mutant. Furthermore, there was no difference in their cell motility as observed on agar motility assay plates (Fig. S2). As the expression of flagella genes is known to be stochastic (Spudich and Koshland, 1976; Korobkova *et al.*, 2004) and as *agn43* homologues are phase variable (Henderson *et al.*, 1997), we propose that the differential expression observed in our RNA‐seq experiment for these genes is likely due to stochastic variation and phase variation, respectively, rather than direct regulation by AggR.

In order to confirm a direct role of AggR in the transcription of genes that showed some of the largest differential expression in our RNA‐seq experiment (*i.e. aafD*, *afaB*, *aap*, *aatP *and *aaiA*), and to identify the AggR‐dependent promoters that control their expression, ~400 bp of upstream DNA was amplified by PCR to generate the *aafD*100, *afaB*100, *aap*100, *aatP*100 and *aaiA*100 promoter fragments (Table S1). Each fragment was cloned into the low copy number *lac* expression vector, pRW50, to generate *lacZ *transcriptional fusions (Table S1) and pRW50 constructs were transferred into the Δ*lac* *E. coli* K‐12 strain, BW25113. To investigate the role of AggR, cells also carried either plasmid pBAD/*aggR*, which encodes AggR expressed from an arabinose‐inducible promoter, or empty pBAD24 vector as a control (Table S1) (Sheikh *et al.*, 2002). Transformants were grown with shaking in LB medium to mid‐logarithmic phase, either with or without AggR induction by arabinose, and measured β‐galactosidase activities were taken as a proxy for promoter activity. Results detailed in Fig. S3 show that for host cells carrying pRW50, containing each of the upstream regulatory region fragments, measured β‐galactosidase levels are higher than levels with empty pRW50, showing that promoter activity is associated with each fragment. Furthermore, expression was markedly increased by arabinose in the presence of pBAD/*aggR*, but not increased with pBAD24. Thus, we conclude that each tested fragment carries an AggR‐dependent promoter, corroborating the results of our RNA‐seq analysis for these promoters.

### Analysis of the AAF/II fimbrial operon promoters from EAEC 042

**Figure 2:**
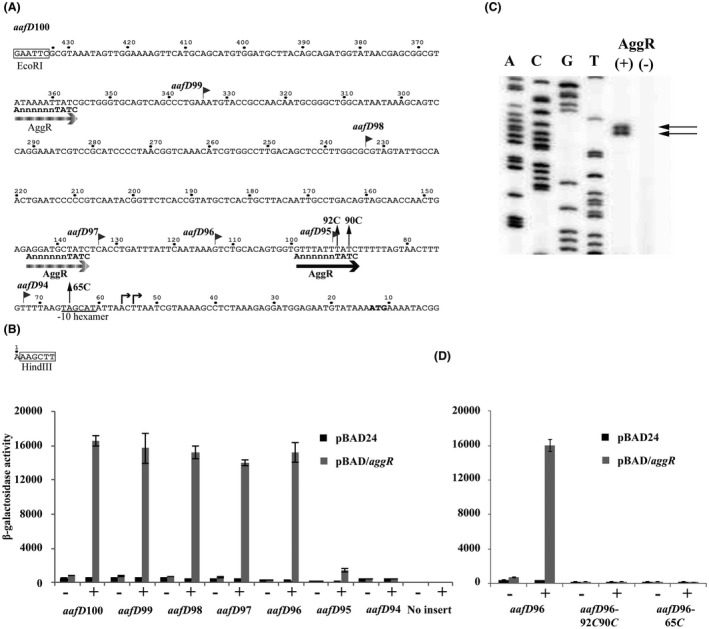
Analysis of the *aafD*100 promoter fragment from EAEC strain 042. A. The panel shows the base sequence of the EAEC 042 *aafD*100 regulatory region fragment, which includes the start of the *aafD* coding sequence. The sequence is flanked by upstream EcoRI and downstream HindIII sites and is numbered from the base immediately upstream of the HindIII site. The limits of the *aafD*99, *aafD*98, *aafD*97, *aafD*96, *aafD*95 and *aafD*94 nested deletions are indicated by flags. The proposed promoter −10 hexamer element is underlined, the experimentally determined transcript start sites are indicated by bent horizontal arrows and the initiating ATG codon is in bold. Potential AggR‐binding sites are indicated by horizontal arrows, with functional and non‐functional sites denoted by dark and light shading respectively. Each site is aligned with the AggR‐binding consensus (Morin *et al.*, 2010). The locations of the 65*C* and 92*C*/90*C* substitutions, which disrupt the −10 element and the functional AggR‐binding site, respectively, are shown. B. The panel illustrates measured β‐galactosidase activities in *E. coli *K‐12 BW25113 ∆*lac* cells, containing pRW50 carrying the *aafD*100 fragment, shortened derivatives or no insert. Cells also carried either pBAD/*aggR *(grey bars) or pBAD24 (black bars). C. The panel shows an autoradiogram of a denaturing polyacrylamide gel run to determine the primer extension products from RNA synthesis initiating at the *aafD* promoter in BW25113 cells carrying pRW50/*aafD*96. AggR (+) and AggR (‐) indicates cells carried pBAD/*aggR *or pBAD24. Reactions are calibrated with the M13mp18 phage reference sequence (A, C, G and T), which serves as sequence ladder. Primer extension products, produced in the presence of AggR, are indicated by arrows. D. The panel shows the β‐galactosidase activities of BW25113 cells, containing pRW50 carrying either the *aafD*96 fragment or mutant derivatives. Cells also carried either pBAD/*aggR *(grey bars) or pBAD24 (black bars). In panels B. and D. cells were grown in LB medium in presence (+) or absence (−) of 0.2% arabinose. β‐galactosidase activities are expressed as nmol of ONPG hydrolysed min^–1^ mg^–1^ dry cell mass. Each activity is the average of three independent determinations and standard deviations are shown for all data points.

**Figure 3:**
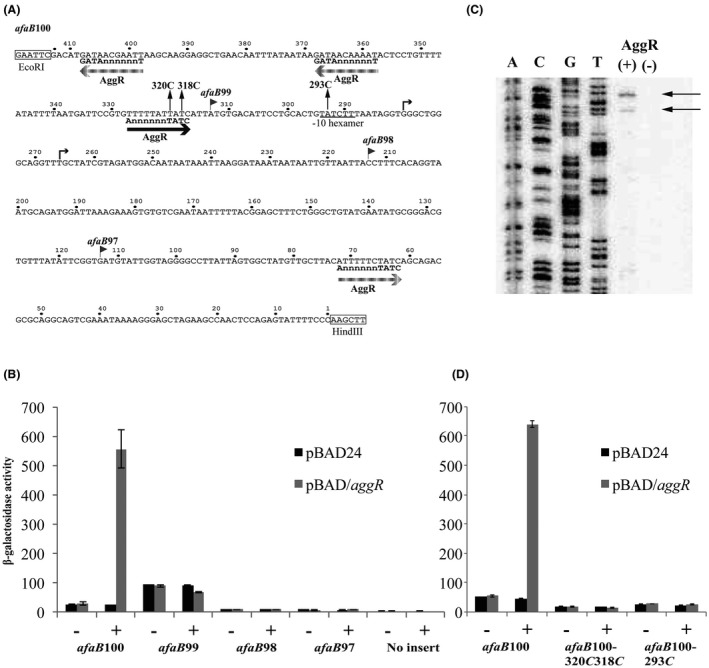
Analysis of *afaB*100 promoter fragment from EAEC strain 042. A. The panel shows the base sequence of the EAEC 042 *afaB*100 regulatory region fragment flanked by upstream EcoRI and downstream HindIII sites. The sequence is numbered from the base immediately upstream of the HindIII site. The limits of the *afaB*99, *afaB*98 and *afaB*97 nested deletions are indicated by flags. The proposed −10 hexamer element is underlined and the experimentally determined transcript start sites are indicated by bent horizontal arrows. Potential AggR‐binding sites are indicated by horizontal arrows, with functional and non‐functional sites denoted by dark and light shading respectively. Each site is aligned with the AggR‐binding consensus (Morin *et al.*, 2010). The location of the 293*C* and 320*C*/318*C* substitutions, which disrupt the −10 element and the functional AggR‐binding site, respectively, is shown. B. The panel illustrates measured β‐galactosidase activities in *E. coli *K‐12 BW25113 cells containing pRW50, carrying the *afaB*100 fragment, shortened derivatives, or no insert. Cells also carried either pBAD/*aggR *(grey bars) or pBAD24 (black bars). C. The panel shows an autoradiogram of a denaturing polyacrylamide gel run to determine the primer extension products from RNA initiating from the *afaB* promoter in BW25113 cells, carrying pRW50/*afaB*100. AggR (+) and AggR (–) indicates cells carried pBAD/*aggR *or pBAD24. Reactions are calibrated with the M13mp18 phage reference sequence (A, C, G and T), which serves as sequence ladder. Primer extension products, produced in the presence of AggR, are indicated by arrows. D. The panel shows the β‐galactosidase activities in BW25113 cells containing pRW50 carrying either the *afaB*100 fragment or mutant derivatives. Cells also carried either pBAD/*aggR *(grey bars) or pBAD24 (black bars). In panels B. and D. cells were grown in LB medium in presence (+) or absence (−) of 0.2% arabinose. β‐galactosidase activities are expressed as nmol of ONPG hydrolysed min^–1^ mg^–1^ dry cell mass. Each activity is the average of three independent determinations and standard deviations are shown for all data points.

During infection, EAEC cells bind to human epithelial cells, using their AAF fimbriae (Harrington *et al.*, 2006). Due to the importance of fimbriae in EAEC pathogenesis and the role of AggR in their expression, we sought to characterize in detail the promoters that control the expression of the EAEC 042 AAF/II fimbrial genes. The AAF/II fimbrial genes are organized into two clusters (*aafDA *and *afaB‐aafCB*) on the pAA2 virulence plasmid (Fig. 1B) (Elias *et al.*, 1999). Figs 2A and 3A detail the DNA sequence of the *aafD*100 and *afaB*100 promoter fragments, which carry DNA upstream of *aafD *and the *afaB *pseudogene respectively. Note that each fragment is flanked by EcoRI and HindIII sites, which were introduced to aid cloning, and sequences are numbered from the HindIII site. Inspection of both sequences identified several matches to the proposed AggR‐binding site consensus (Morin *et al.*, 2010) (Figs 2A and 3A). Therefore, to identify the essential sequences for AggR‐induced promoter activity, we initially focused on the *aafD*100 promoter fragment and constructed nested deletions from the EcoRI end of the fragment. Each shortened fragment (*i.e. aafD*99, *aafD*98, *aafD*97, *aafD*96, *aafD*95 and *aafD*94) (Fig. 2A, Table S1) was cloned into pRW50 and each plasmid construct was transferred into BW25113 cells, carrying pBAD/*aggR* or pBAD24. β‐galactosidase activity was measured, as before, and results in Fig. 2B show that *aafD*96 is the shortest fragment where full AggR‐dependent induction is retained, with induction being greatly reduced for *aafD*95 and absent for *aafD*94. To identify the transcript start, we extracted RNA from BW25113 cells carrying pRW50/*aafD*96 and containing either pBAD/*aggR* or pBAD24. Fig. 2C shows the result of the primer extension analysis, analysed by polyacrylamide gel electrophoresis, and identifies two clear bands, corresponding to transcripts starting at positions 56 and 54 of the cloned sequence (Fig. 2A). Note that these bands are seen in the sample from cells carrying pBAD/*aggR* but not with pBAD24. Examination of the *aafD*96 DNA sequence upstream of positions 56 and 54 revealed a potential −10 hexamer element (5′‐TAGCAT‐3′) and a potential AggR‐binding site (5′‐GTTTATTTATC‐3′), based on previously established consensus sequences (Morin *et al.*, 2010; Browning and Busby, 2016) (Fig. 2A). Therefore, to investigate the role of these sequences, site‐directed mutagenesis was used to introduce the 65*C* and 92*C*/90*C* substitutions into the *aafD*96 fragment, to disrupt each element (Fig. 2A). Mutant derivatives were cloned into pRW50, transferred into BW25113 cells, carrying pBAD/*aggR* or pBAD24, and the promoter activity determined. Results in Fig. 2D show that AggR‐dependent promoter activity from the *aafD96 *fragment was greatly decreased by these substitutions, consistent with our proposal of these elements as the −10 hexamer and AggR‐binding site at the *aafD *promoter.

To locate the essential promoter sequences required for *afaB‐aafCB* expression, we also constructed nested deletions of the *afaB*100 promoter fragment (Fig. 3A). Again, each of the shorter fragments (*afaB*99, *afaB*98 and *afaB*97) (Fig. 3A, Table S1) was cloned into pRW50 and promoter activity determined. Results in Fig. 3B show that *afaB*100 is the only fragment where AggR‐dependent induction is observed. Thus, to identify the start of transcription, we again extracted RNA from BW25113 cells carrying pRW50/*afaB*100 with either pBAD/*aggR* or pBAD24. Fig. 3C shows the result of the primer extension analysis and identifies two bands, corresponding to transcripts starting at positions 280 and 266 of the cloned sequence (Fig. 3A), which were only present in the sample from cells carrying pBAD/*aggR*. Examination of the *afaB*100 DNA sequence upstream of these positions revealed a potential −10 element (5′‐TATCTT‐3′) and AggR‐binding site (5′‐TTTTTATTATC‐3′) (Fig. 3A). These elements were, therefore, disrupted by introducing the 293*C* and 320*C*/318*C* substitutions into the promoter region (Fig. 3A) and mutant *afaB*100 fragments were cloned into pRW50. As expected, AggR‐dependent promoter activity was substantially decreased by these substitutions (Fig. 3D), consistent with our hypothesis that these sequences constitute a functional −10 element and AggR‐binding site at the *afaB *promoter. Previously, Elias *et al. *(1999) predicted that the promoter controlling *aafCB* expression was immediately upstream of *aafC*. Therefore, using our dual reporter system, we checked for promoter activity in different *afaB‐aafCB* fragments. However, we could not find any evidence of a second promoter (Fig. S4). Thus, our results with both *aafD *and *afaB *promoter fragments indicate that each EAEC 042 AAF/II fimbrial gene cluster is expressed from a single upstream AggR‐dependent promoter.

### Other EAEC fimbrial operon promoters possess similar promoter organization

**Figure 4:**
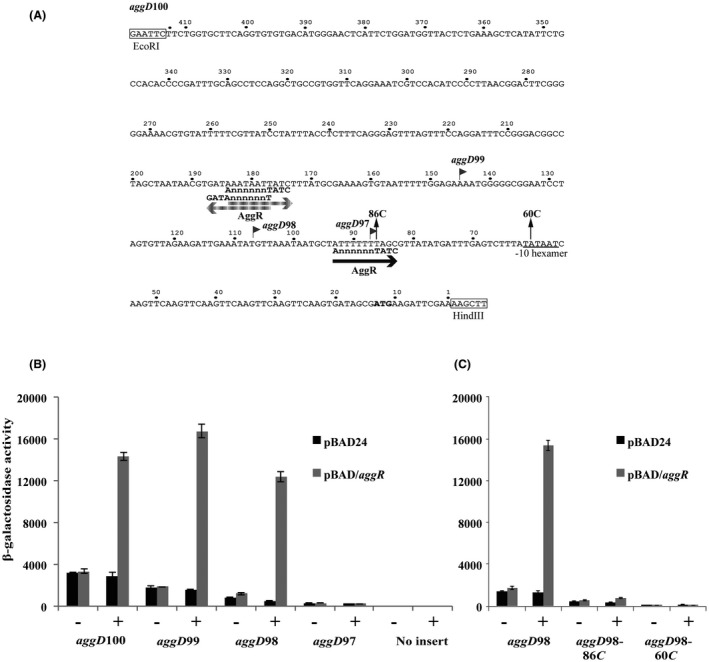
Analysis of *aggD*100 promoter fragment from EAEC strain 17‐2. A. The panel shows the base sequence of the EAEC 17‐2 *aggD*100 regulatory region fragment, which includes the start of the *aggD* coding sequence. The sequence is flanked by upstream EcoRI and downstream HindIII sites and is numbered from the HindIII site. The limits of the *aggD*99, *aggD*98 and *aggD*97 nested deletions are indicated by flags. The proposed −10 hexamer element is underlined and the initiating ATG codon is in bold. Potential AggR‐binding sites are indicated by horizontal arrows, with functional and non‐functional sites denoted by dark and light shading respectively. Each site is aligned with the AggR‐binding consensus (Morin *et al.*, 2010). The location of the 60*C* and 86*C* substitutions, which disrupt the −10 element and the functional AggR‐binding site, respectively, is shown. B. The panel illustrates measurements of β‐galactosidase expression in *E. coli *K‐12 BW25113 ∆*lac* cells, containing pRW50 carrying the *aggD*100 fragment, shortened derivatives or no insert. The cells also carried either pBAD/*aggR *(grey bars) or pBAD24 (black bars). C. The panel shows the β‐galactosidase activities of BW25113 cells containing pRW50 carrying either the *aggD*98 fragment or mutant derivatives. Cells also carried either pBAD/*aggR *(grey bars) or pBAD24 (black bars). In panels B. and C. cells were grown in LB medium in presence (+) or absence (−) of 0.2% arabinose. β‐galactosidase activities are expressed as nmol of ONPG hydrolysed min^–1^ mg^–1^ dry cell mass. Each activity is the average of three independent determinations and standard deviations are shown for all data points.

To date, five AAF systems (AAF/I to AAF/V) have been identified in EAEC strains and the genes that encode these fimbrial components, together with the corresponding chaperones and ushers, are all found on large virulence plasmids (Savarino *et al.*, 1994; Elias *et al.*, 1999; Bernier *et al.*, 2002; Boisen *et al.*, 2008; Jonsson *et al.*, 2015). As the promoters that control the expression of different AAF variants have not been characterized, we investigated some of these promoters in more detail to uncover their promoter organization and determine whether AggR regulates them similarly. It has been shown that EAEC strain 17‐2 and the highly virulent Shiga‐toxin‐producing EAEC O104:H4 strain C227‐11, produce AAF/I fimbriae, and the fimbrial genes are organized in a single operon (*aggDCBA*) (Savarino *et al.*, 1994; Rasko *et al.*, 2011; Rohde *et al.*, 2011). Therefore, to identify the fimbrial operon promoter from EAEC 17‐2, PCR was used to amplify the DNA upstream of *aggD* to generate the *aggD*100 promoter fragment (Fig. 4A). This was cloned into pRW50 and assayed for promoter activity in BW25113 cells, carrying either pBAD24 or pBAD/*aggR*, as before. Results detailed in Fig. 4B show that expression from *aggD*100 fragment was greatly increased by AggR induction, confirming that the EAEC 17‐2 *aggD *promoter is AggR regulated. To pinpoint the location of important regulatory sequences, nested deletions were constructed and the shortened fragments (*i.e. aggD*99, *aggD*98 and *aggD*97) were cloned into pRW50 and assayed (Fig. 4). Results in Fig. 4B show that AggR‐mediated induction is absent with the *aggD*97 fragment, and that *aggD*98 is the shortest of the fragments where AggR‐dependent promoter activity is observed. Examination of the *aggD*98 DNA sequence revealed a potential promoter −10 element (5′‐TATAAT‐3′) and an AggR‐binding site (5′‐ATTTTTTTAGC‐3′) (Fig. 4A). Disruption of these elements in the *aggD*98 fragment, by introducing the 60*C* and 86*C* substitutions, respectively, greatly decreased promoter expression (Fig. 4C), supporting our proposal that these are the functional −10 element and AggR‐binding site at this promoter.

Savarino *et al. *(1994) noted that the EAEC 17‐2 AAF/I *aggD *promoter carried six direct repeats of the hexamer 5′‐TCAAGT‐3′, which are positioned between the −10 element and the *aggD* translation initiation codon (Fig. S5). Interestingly, these repeats are more extensive in the *aggD *promoters from other pathogenic EAEC strains, *e.g.* the EAEC O104:H4 strain C227‐11 possesses 15 repeats (Table S5 and Fig. S5). As such tandem repeats are unusual in bacteria and can play a role in gene expression (Browning and Busby, 2016), we examined if the different number of repeats carried by the EAEC 17‐2 and C227‐11* aggD *promoters affected promoter activity. Results detailed in Fig. S5 show that the two promoters had similar promoter activity and, thus, although these repeat tracts are substantial, they do not appear to influence *aggD* promoter activity.

**Figure 5:**
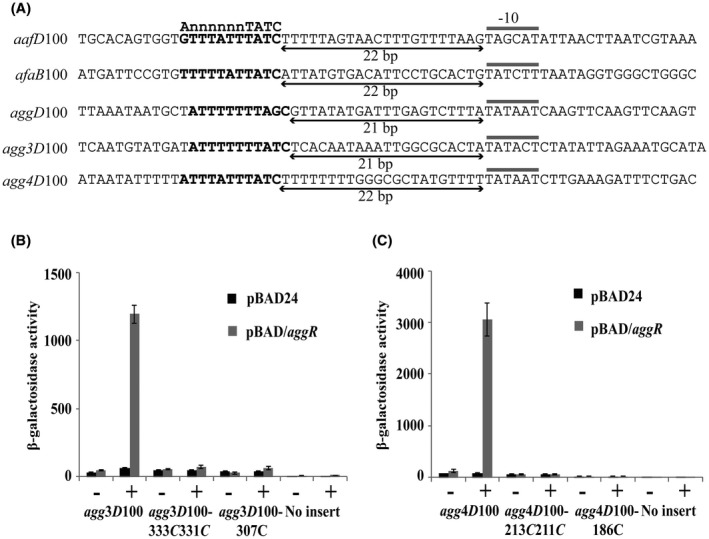
Analysis of EAEC 55989 *agg3D*100 and EAEC C1010‐00 *agg4D*100 promoter fragments. A. The panel shows the sequences of AggR‐dependent fimbrial promoters investigated in this study. The AggR‐binding sites are bold type and the −10 hexamer elements are indicated by grey lines. The underline double arrowheads mark the distance between AggR‐binding sites and −10 hexamer elements. B. The panel illustrates the β‐galactosidase activities of BW25113 cells containing pRW50 carrying various *agg3D*100 and *agg4D*100 promoter derivatives, from EAEC strains 55989 and C1010‐00. Cells also carried either pBAD/*aggR *(grey bars) or pBAD24 (black bars) and were grown in LB medium in presence (+) or absence (−) of 0.2% arabinose. β‐galactosidase activities are expressed as nmol of ONPG hydrolysed min^–1^ mg^–1^ dry cell mass. Each activity is the average of three independent determinations and standard deviations are shown for all data points. The 307*C* and 331*C*/333*C* substitutions disrupt the −10 element and AggR‐binding site, respectively, in the EAEC 55989 *agg3D*100 promoter fragment, whilst the 186*C* and 211*C*/213*C* substitutions disrupt the corresponding sequences in the EAEC C1010‐00 *agg4D*100 fragment (see Fig. S4).

Alignment of the nucleotide sequence of the AAF/I and AAF/II fimbrial operon promoters (Fig. 5A) indicated that in each case, the DNA‐binding site for AggR is located 21 or 22 bp upstream from the −10 element. This suggests that all AggR‐dependent fimbrial promoters may have similar promoter organization. Using this information, we examined the DNA upstream of the *agg3D *and *agg4D *genes, which are the first genes in the AAF/III and AAF/IV fimbrial operons from the pathogenic EAEC strains 55989 and C1010‐00, respectively, and identified suitably positioned AggR‐binding sites and −10 elements (Bernier *et al.*, 2002; Boisen *et al.*, 2008) (Figs 5A and S6). To investigate the regulation of these AAF variants, the DNA upstream of *agg3D *and *agg4D* was cloned into plasmid pRW50, to generate the *agg3D*100 and *agg4D*100 promoter fragments, and point mutations were introduced to disrupt the potential AggR‐binding sites and −10 elements identified (Figs 5A and S6). The β‐galactosidase activity of BW25113 cells, carrying these constructs, was then measured, as before. Results in Fig. 5B and 5C indicated that expression from both the wild‐type *agg3D*100 and *agg4D*100 fragments, respectively, is dependent on AggR and that disruption of the proposed AggR‐binding sites and −10 elements, in each fragment, completely abolished promoter activity. Thus, we have identified important elements controlling *agg3D *and *agg4D *expression and our results are in agreement with a common promoter organization existing for many EAEC AggR‐dependent promoters.

### AggR‐dependence can be conferred by a single correctly positioned AggR‐binding site

**Figure 6:**
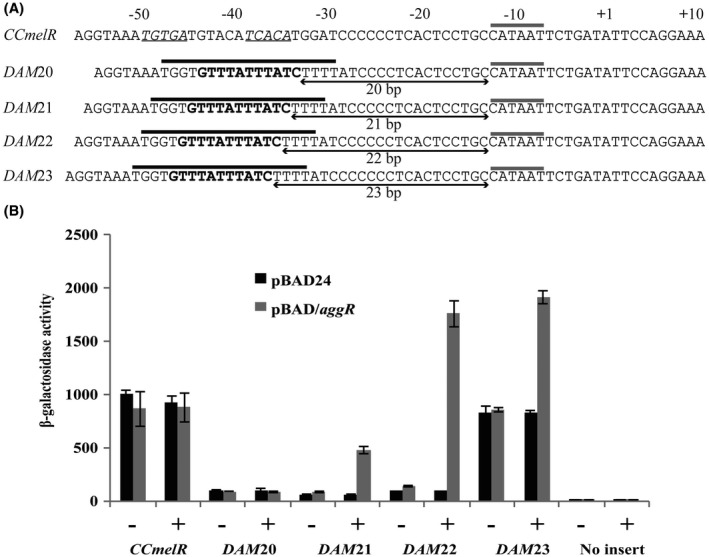
Construction and analysis of semi‐synthetic AggR‐dependent promoters. A. The panel ilustrates the DNA sequence of the *CCmelR* promoter region and the *DAM*20, *DAM*21, *DAM*22 and *DAM*23 promoter constructs. In these promoters, the AggR‐binding site from the *aafD *promoter has been transplanted at different distances from the −10 elements (20 bp to 23 bp). The CRP‐binding half‐sites in the *CCmelR* promoter are italicized and underlined*.* Thick black lines indicate the *aafD* promoter sequence transplanted, with the AggR‐binding site in bold, and the −10 elements are indicated by grey lines. Sequence is numbered from the *CCmelR* promoter transcript start site (+1)*. *B. The panel illustrates the measured β‐galactosidase activities in BW25113 cells, containing pRW50 carrying the* CCmelR *and various *DAM *promoter derivatives. Cells also carried either pBAD/*aggR *(grey bars) or pBAD24 (black bars). Cells were grown in LB medium in presence (+) or absence (−) of 0.2% arabinose. β‐galactosidase activities are expressed as nmol of ONPG hydrolysed min^–1^ mg^–1^ dry cell mass. Each activity is the average of three independent determinations and standard deviations are shown for all data points.

Our data suggest that a single correctly positioned AggR‐binding site may be all that is required to confer AggR‐dependent regulation on target promoters. To test this, we generated a series of semi‐synthetic promoters in which the functional AggR‐binding site from the *aafD *promoter was transplanted into the well characterized *E. coli* *melR *promoter, known to be dependent on activation by the cyclic AMP receptor protein (CRP) (Webster *et al.*, 1988). To do this, we used the previously constructed *CCmelR* promoter, which carries a consensus DNA site for CRP. Fig. 6A shows the base sequence of the promoter elements in the resulting fragments, denoted *DAM*20 to *DAM*23, where the *melR *promoter CRP site is replaced by a DNA site for AggR, located 20 to 23 bp upstream from the *melR* promoter −10 element (5′‐CATAAT‐3′). These fragments, together with the *CCmelR* fragment, were cloned into pRW50. BW25113 cells, containing either pBAD/*aggR* or pBAD24, were transformed with these recombinant plasmids and promoter activities were determined. The β‐galactosidase activity measured in cells containing pRW50/*CCmelR* and pRW50/*DAM*20 showed no increase on induction of AggR expression (Fig. 6B). However, measured activity in cells containing pRW50/*DAM*21, pRW50/*DAM*22 and pRW50/*DAM*23 showed a four‐, eight‐ and twofold increase in expression levels, respectively, compared to the control without AggR (Fig. 6B). Thus, we conclude that transplanting a single AggR‐binding site into a promoter can confer AggR‐dependence and that a spacing of 22 bp between the DNA site for AggR and the −10 element is optimal for induction.

## Discussion

Using an RNA‐seq approach, we identified the genes regulated by AggR in the archetypal pathogenic EAEC strain 042. Some members of the AggR regulon are conserved hypothetical genes with unknown function and their regulation by AggR suggests a possible role in EAEC 042 intestinal colonization. As EAEC strains are heterogeneous in nature, further investigation of these candidate genes has the potential to enhance our knowledge of EAEC pathogenicity.

The main aim of this study was to determine the organization and architecture of AggR‐dependent promoters. Focusing on the fimbrial operon promoters in EAEC strain 042, we found single AggR‐dependent promoters upstream of the *aafDA *and *afaB‐aafCB* regions on the pAA2 virulence plasmid that encode genes for fimbrial assembly. The *aafD* promoter is located immediately upstream of the *aafD* gene, which encodes the AAF/II chaperone protein, whilst the *afaB* promoter is upstream of the *afaB* pseudogene, which is followed by functional *aafC* and *aafB* genes, encoding the fimbrial usher protein and the fimbrial adhesin respectively (Figs 1B, 2A, 3A and S4). It is of note that the level of expression from the *aafD *promoter is considerably higher than that of *afaB *(Figs 2 and 3) with the fold increase in RNA sequence reads for *aafDA *genes being higher than that of the *aafCB* genes (Table S2). As the *aafD *promoter controls the expression of the AafD chaperone and AafA fimbrial subunit, both of which are required in large amounts, in comparison to the AafC usher protein and AafB adhesin, this regulation is likely to help ensure that each component of the AAF/II fimbriae are made to the appropriate level. For the other AAF systems examined (*e.g *AAF/I, AAF/III and AFF/IV) the fimbrial genes exist in a single operon and were expressed from a strong upstream AggR‐regulated promoter that had similar organization to the EAEC 042 fimbrial promoters (Fig. 5A).

**Figure 7:**
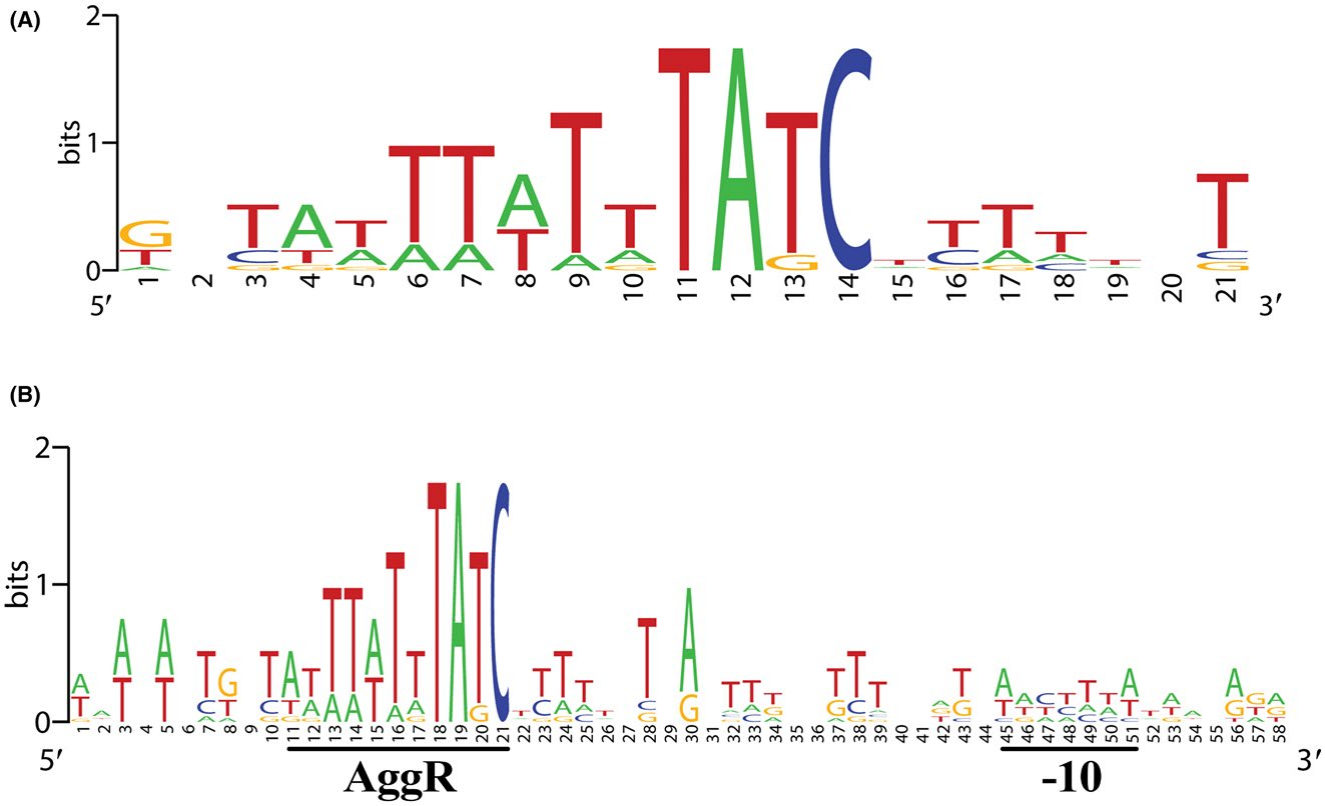
The AggR‐binding site consensus. The figure shows motifs for: A. the AggR‐binding site consensus sequence and B. AggR‐dependent promoter organization. Motifs were generated using the WebLogo server (Crooks *et al.*, 2004) with sequences from the EAEC 042 *aafD *and* afaB* promoters, the EAEC 17‐2 *aggD* promoter, the EAEC 55989 *agg3D* promoter and the EAEC C1010‐00 *agg4D *promoter identified by experiments in Figs. 2, 3, 4, 5, the *aap*,* aatP *and *aaiA *promoters identified by similar experiments by Yasir (2017) and the AggR‐binding site at the *aggR *promoter (Morin *et al.*, 2010).

Previous studies have shown that DNA sites for AggR‐binding resemble sites for the Rns ‘master’ regulator from enterotoxigenic *E. coli* (ETEC) (Munson, 2013) and, following studies of the *aggR* promoter, a consensus sequence was also suggested for AggR, in which the importance of a TATC motif and an A base, seven nucleotides upstream of this motif, was highlighted (Morin *et al.*, 2010). Thus, we first identified putative DNA sites for AggR using this consensus. Based on the promoters characterized here, we now propose a revised consensus logo for the AggR‐binding site and AggR‐dependent promoters (Fig. 7). Note that, in our AggR‐binding site consensus the upstream A base, noted by Morin *et al. *(2010), is not always conserved (Figs 5A and 7A). Indeed, using the *aafD *promoter from EAEC 042, which has a G at this position, we observed that any base can be tolerated at this position, with only a small effect on AggR‐dependent activation (Fig. S7). For each fimbrial promoter, we found a single functional DNA site for AggR, located 21 to 22 base pairs upstream from the promoter −10 element and this juxtaposition suggests that bound AggR must overlap the −35 element and is able to interact directly with Domain 4 of the RNA polymerase σ subunit. This is consistent with AggR being a member of the AraC‐XylS family of bacterial transcription factors, many of which activate transcription initiation by making such a direct contact that serves to assist the recruitment of RNA polymerase to the target promoter (Martin and Rosner, 2001; Egan, 2002; Browning and Busby, 2004). It is also evident from our promoter logo that the DNA between AggR‐binding site and the −10 element contains phased A/T tracts (Fig. 7B), which is indicative of bent DNA. Indeed, modelling of the *aggD, aafD *and *afaB *promoters suggests that AggR‐dependent promoters possess a bent promoter architecture (Fig. S8). Our experiment, where a single DNA site for AggR was ‘transplanted’ into the context of the *E. coli melR* promoter, indicates that it is easy for AggR‐dependence to be conferred onto a target promoter (Fig. 6). Since AggR‐binding sites are relatively degenerate, essentially consisting of a conserved TATC motif with an upstream A/T tract, it may be simple for the promoters expressing A/T‐rich horizontally acquired genes, to become AggR‐dependent and assimilated into the AggR regulon.

As our transcriptomics data identified a number of genes that had not previously been included in the AggR regulon (Fig. 1, Tables S2 and S3) (Morin *et al.*, 2013) we used the information from our AggR‐dependent promoter logo (Fig. 7B) to interrogate the genes identified in our RNA‐seq data set. Thus, we were able to find AggR‐binding sites and suitably positioned −10 promoter elements (with a spacing of 21 to 23 bp) upstream of many leading genes in the transcription units that we found to be AggR regulated on both pAA2 and the chromosome (Tables 1 and 2, respectively). This analysis confirmed the organization of AggR‐dependent promoters characterized by this study (*i.e. aggR, aatP, aap *and *aaiA*) and it is of note that these promoter sequences are conserved in other pathogenic EAEC strains, *e.g.* C227‐11 and 55989, suggesting that these genes are similarly regulated in these strains (Fig. S9).

**Table 1:** Potential AggR‐dependent promoters located on plasmid pAA2 from EAEC strain 042.

Gene name	Promoter sequence^a^	Spacing (bp)^b^	Distance to start (bp)^c^
EC042_pAA003	CAGCAATTAACTATTGCT**ATAATAATATC**TATTATTTTTTTTGTTTTGATT**TAT** C **AT**TTAATTTTTTATAGATAA	22	101
EC042_pAA003	ATCTATTATTTTTTTTGT**TTT** G **ATTTATC**ATTTAATTTTTTATAGATAAAA**TA** AC **TT**TTTGGTTTTTAATATAGT	22	75
EC042_pAA005A	CAGCAATCAACTATTGCT**ATAATAATATC**TATTTTTTGATTTATTTTGTTT**TA** GC **AT**GTAAAAGTTTAGAAAAAG	22	56
EC042_pAA006	TGTATATCAAAGAGTCAG**TAATAATTATC**CCTATAAAGCTAAGAGATAAAAA **A** AC **A** AGAAATGCAAAATTCTGCC	22	481
*aatP* d	TTATTATCATAAACTTAT**A** G **TTATATATC**CCTTAGTTATTAATAGTTGGG**TA** C **A** T **T**ATATAGTGTTTCCAATAAC	21	100
EC042_pAA019	ACATAAAAAGTGATCATGGC **AATAATATC**CGGAAATCTAGTAACTATGCTAA **AT** T **A** CAGGATGACTGTTTTTCTA	22	370
EC042_pAA019	TAATTAAGCAATACTCAT**AAAATTATATC**AAATATGATTTTTAGCTGTAA**TA** AC **AT**TATCAGATGGAACGTGCAG	21	47
*afaB* d	TATTTTAATGATTCCGTG**TTTTTATTATC**ATTATGTGACATTCCTGCACTG**TAT** CT **T**TAATAGGTGGGCTGGGCA	22	303
EC042_pAA033	CTAAATGCCTTCAATATA**TT** G **TATATATC**AACCAATATGATAAAGTTATTCG**TAT** T **AT**TAACATTCAGAACAATC	23	415
*aafD* d	TAAAGTCTGCACAGTGGTG **TTTATTTATC**TTTTTAGTAACTTTGTTTTAAG**TA** GC **AT**ATTAACTTAATCGTAAAA	22	75
EC042_pAA051	TTCAAGAATTACTTCAGA**TTT** G **ATATATC**TTATATCAAAGATCGAAAACAAC **A** A **AA** AAATTATAGAGTCAATTTA	22	493
EC042_pAA051	ATTACTTCAGATTTGATA**TAT** C **TTATATC**AAAGATCGAAAACAACAAAAAA **AT** T **AT**AGAGTCAATTTATATATCG	21	486
EC042_pAA051	AAAAAAATTATAGAGTCA**ATTTATATATC**GGCTGTAAGCTTCTTTTC**TG**A**TA** A **A** G **T**CAGAAACATAATCGAGAAA	21	441
EC042_pAA051	TAAAAGCCAATAAAAACA**T** G **TTT** C **ATATC**ATTATTTGAGATTGCTATAAAC **ATA** T **T**GAGATGGCTGAAGTTGGTG	21	97
*aggR * d	ACGTATTTTATATGAGTT**AAAAATATATC**TTTTTATTGATAAGAGTTAGGTC **AT** TC **T**AACGCAGATTGCCTGATA	22	73
EC042_pAA053	GACAAATAAGGTTGGAGTCC **AAAAATATC**GTGCTCTAACCGAATGGGTTAA **ATAAT**ATCTAGCTCTAGCTAAAAT	21	314
*aap * d	ACACTCGATATATGTTGC**TATTTTTTATC**TGGCCGCAACTCTTATTTA**TG**C**TA** GCC **T**TCTAAAAGGAGGGGCGGC	22	134
*aar*	AGGGGGTCAGCTCACAAA**ATAAA** GG **TATC**TTCCAGCACGGAGCAATAGACG**TA** AC **A** ATCACTTAAAAAAACGGAG	22	179
*aar*	AAAAATCTACATTGTGTC**ATTATT** C **TATC**CTTCCGATATCTTATCATGTTA**TA** A **A** T **T**CCAGAAAAGAGAACATTG	22	48
EC042_pAA061	AGTGGCTGCGTTACGTCA**TT** G **AA** C **ATATC**CAGGACTGGCCGGCAAACCGGG**TA** CGCGATCTGTTGCCCTGGAAAG	22	161
EC042_pAA061	AAAGTTGATCTGAGCTCTC **A** G **TAAATATC**AATACGGTTCTGACGAGCCGCT**TA** CC **A** GCGACCAATCGATGAACGG	22	90

a Promoters were aligned to the AggR‐binding site consensus (WWWWWWWTATC), only allowing a maximum of two mismatches within the A/T tract region, and the extended −10 region consensus (TGnTATAAT). Each element is underlined and matches to each consensus are bold.

b Distance between the AggR‐binding site and the −10 hexamer element.

c Distance between the AggR‐binding site and the predicted translational start site of each gene.

d Characterized AggR‐dependent promoter.

**Table 2:** Potential AggR‐dependent promoters located on the chromosome of EAEC strain 042.

Gene name	Promoter sequence^a^	Spacing (bp)^b^	Distance to start (bp)^c^
*bssS*	GTACGTTATGCCGCAGCG**AATAATTTATC**GGTTATTGGCGCAACAAAAGAAG **ATAA** ACAGCGCATTAGCGAAATT	22	383
*flgB*	TGTGGCCTGCACATTAACC **T** G **TAAATATC**GTTTGTCGTTACCGCAGCG**TG**CC **A** AC **A** CATTCACATTGCCCCACAG	22	408
*cspI*	ATGGTGTTCTGGTTTGTT**A** C **AAATTTATC**TGAAGCAGTCATTGTTATAATTT**TAT** T **AT**TTGTACCTCTTGAGATT	23	124
*fliE*	ACTGGTAGGCTTTGCTACC **A** G **AAATTATC**CGGGAGATGAAAATGTCAGCGA**TA** C **A** GGGGATTGAAGGGGTTATCA	22	12
EC042_2219	TCTCCTTATACTAAAGAA**ATAAT** C **ATATC**AAAATAAAAATTCACAACAG**TG**CA **A** C **A** T **T**AAAAAATACAACCAACA	23	491
EC042_2219	GGAGATAATATTATGTCA**AAAAAAATATC**AGCCATTGCTTATAACTCATTA**TAT** GTCAACATGGGTCACTATAAC	22	5
EC042_2249	CTTAGCCGATTTTCTGTA**A** GG **ATTTTATC**GTGTCAGACACACTCCCCGGGACA **A** C **A** C **T**TCCCGACGACAATCACG	23	457
EC042_2249	TACCCTCAGCAGTTGTGG**TTA** CG **TTTATC**TGGCTGTTTATCCGACGCCCGAA **AT** G **A** AAAATTAACTCTCCAGAAT	22	123
EC042_2249	TCACCACGCCACTTTTCC**ATTTTTATATC**TGCATATCAGGAAAATCTTCAG**TAT** G **A** AAACATTACCTGTATTACC	22	23
EC042_3188	TCACTGACACTGATGTCTG **TTTTATTATC**GCCTTTATCTCTTCAGGCCGCGG **ATA** CCAGATCACGCAGAGTATGA	22	24
EC042_4006	TGATGTGTTGTCAGTTTC**A** C **TTTTTTATC**CTTTTTTTAATCGTTAACTGAC**TATAAT**GGCAAGATCACTACGATT	22	200
EC042_4430	TTTGTTTCATTAATTTTG**T** G **AA** C **TATATC**ACAATTGATTGTTTGTTAGCCAG **AT** T **A** GGCCGTGACTTTTATTGCC	22	286
EC042_4430	ATTCCTGTAGGAAATTAG**TTTT** G **AATATC**AATGAATTATTTTTATTCAGGTG **A** CG **AT**TAAAAAGGTATCAATTTC	22	154
EC042_4430	TTTATTCAGGTGACGATT**AAAAA** GG **TATC**AATTTCAAATCAGGCAAAAG**TG**C**TAT** TTATACCGTAAGATTTATCT	23	114
*aaiA* d	GTTATATCATTAATCAGC**AAAAAT** G **TATC**ACATGCTCACTTTCTTTTTA**TG**G**TAT** C **A** CTATATAGAATCCATGAA	23	237

a Promoters were aligned to the AggR‐binding site consensus (WWWWWWWTATC), only allowing a maximum of two mismatches within the A/T tract region, and the extended −10 region consensus (TGnTATAAT). Each element is underlined and matches to each consensus are bold.

b Distance between the AggR‐binding site and the −10 hexamer element.

c Distance between the AggR‐binding site and the predicted translational start site of each gene. Sequence corresponding to the open reading frame of each gene is in red, where applicable.

d Characterized AggR‐dependent promoter.

AggR‐dependent biofilm formation is a hallmark of EAEC infection and, in addition to the expression of AAF fimbriae, other plasmid‐encoded genes are required (*e.g. *EC042_pAA003, EC042_pAA004 and *shf*) (Czeczulin *et al.*, 1997; Fujiyama *et al.*, 2008; Morin *et al.*, 2013). Our analysis indicates chromosomally encoded genes, EC042_4006 (*yicS*) and *bssS*, are also AggR regulated (Tables 2 and S3). Both genes have been implicated in biofilm formation in *E. coli*, whilst YicS plays a role in pathogenicity in avian pathogenic *E. coli *(Domka *et al.*, 2006; Verma *et al.*, 2018). Thus, it is likely that AggR deploys both specialized plasmid‐ and chromosomally‐encoded factors to ensure formation of its trademark biofilm.

Strikingly, AggR appears to control the expression of a number of transposases and transposon remnants (Tables S2 and S3) and putative AggR‐dependent promoters are located upstream of these transcription units (Tables 1 and 2). This suggests that genomic rearrangements, on both the chromosome and the pAA2 plasmid, may occur more frequently in EAEC 042 during the initiation of the AggR virulence programme and lead to genome evolution, something which has been observed in other bacterial species (Lindsay, 2014; Singh *et al.*, 2014; Wan *et al.*, 2017).

AggR belongs to a subgroup of AraC‐XylS family members, which control virulence gene regulation, and includes Rns/CfaD/CfaR from ETEC and VirF from *Shigella flexneri*. These family members are highly similar and often interchangeable, for example Rns can replace VirF in *S. flexneri* and CfaR can complement for the loss of AggR in EAEC (Caron and Scott, 1990; Nataro *et al.*, 1994; Porter *et al.*, 1998). However, it is worth noting that this arrangement is not always reciprocal, as VirF is unable to replace Rns in ETEC, and this might reflect subtle differences in the mechanisms by which each regulator activates transcription (Porter *et al.*, 1998). As well as directly activating transcription, both Rns and VirF have been shown to activate at promoters by counteracting the repressive effects of the heat‐stable nucleoid structuring protein, H‐NS, which silences many horizontally acquired genes (Jordi *et al.*, 1992; Tobe *et al.*, 1993; Murphree *et al.*, 1997; Singh *et al.*, 2016). Experiments, which examined AggR‐dependent activation at the *afaB *and *aafD *promoters in an *hns *null strain (Fig. S10), indicated that, although H‐NS marginally represses both promoters, AggR still substantially activates transcription in the absence of H‐NS. Consistent with this, a recent transcriptomic analysis in EAEC 042 indicated that neither *afaB *nor *aafD* were derepressed by the absence of H‐NS or its homologue H‐NS2 (EC042_2824) (Prieto *et al.*, 2018). Therefore, we propose that, at the *afaB *and *aafD* promoters, AggR primarily activates transcription by directly interacting with RNA polymerase rather than alleviating H‐NS repression.

To characterize AggR‐dependent promoters, we, as have others, used a simple two‐plasmid system with a laboratory strain of *E. coli* K‐12 as host (Dudley *et al.*, 2006; Morin *et al.*, 2010). As expected for promoters that control the expression of virulence determinants, coupling of expression to AggR is tight, with high induction ratios. For some AraC‐XylS family members that control bacterial virulence, specific host‐derived signals are often sensed by the protein, which modulates the transcription factors activity (Yang *et al.*, 2009; Childers *et al.*, 2011). However, neither temperature nor specific molecules, such as bicarbonate ions or bile salts, seem to play a major role in AggR‐dependent activation (Morin *et al.*, 2013) (Table S6). Thus, it is unclear what signal, if any, is sensed by AggR, especially as we were able to observe AggR‐dependent activation in laboratory *E. coli* K‐12, without any special induction conditions. It is of note that in EAEC, AggR activity is controlled by the Aar repressor protein (Santiago *et al.*, 2014), which could explain why we were able to detect AggR‐dependent activity in its absence. Thus, it is clear that understanding the signal and mechanism by which the AggR‐mediated regulation is initiated in EAEC strains will be key to understanding and designing small molecule inhibitors which can short circuit virulence in this important *E. coli *pathotype.

## Experimental procedures

### Bacterial strains, plasmids, primers and growth conditions

The bacterial strains, plasmids and promoter fragments used in this study are listed in Table S1. The oligonucleotide primers used for primer extension analysis and to amplify and mutate the various DNA fragments are listed in Table S7. Standard procedures for PCR, cloning and DNA manipulation were used throughout (Sambrook and Russell, 2001). All DNA fragments used in this study are flanked by EcoRI and HindIII sites and the DNA sequence of each fragment is numbered from the base adjacent to the HindIII site. Base substitutions are defined by the position of the nucleotide base altered and the substituted base introduced. Cells were routinely grown in Lysogeny Broth (LB medium) at 37°C with shaking. To measure promoter activities, fragments were cloned into the *lac *expression vector pRW50 (Lodge *et al.*, 1992) and maintained with 15 μg ml^–1^ tetracycline. To examine the effect of *aggR *expression, cells were transformed with either pBAD/*aggR* or pBAD24, which were maintained in cells with 100 µg ml^–1^ ampicillin or carbenicillin. AggR expression, using pBAD/*aggR*, was induced by the inclusion of 0.2% w/v arabinose in the medium, where appropriate (Sheikh *et al.*, 2002).

### RNA isolation, rRNA depletion and cDNA synthesis for RNA‐seq

Triplicate overnight cultures of EAEC 042 and EAEC 042 Δ*aggR *were used to inoculate 50 ml of Dulbecco’s modified Eagle’s medium with 0.45% glucose (DMEM high glucose) (Sigma) to an OD_600_ of 0.05. Cultures were grown at 37°C with shaking to an OD_600_ of 0.6. RNA was isolated using an RNeasy Mini Kit (Qiagen) and contaminating DNA was removed using an RNase‐free DNase kit (Qiagen). The quality of the RNA was checked using an Agilent RNA 6000 Nano Chip (Agilent Technologies). RNA samples with a RIN (RNA integrity number) above 8 were then used for RNA‐seq. A total of 3.5 μg of isolated RNA was used for each sample for rRNA depletion using a Ribo‐Zero™ rRNA Removal Kit for bacteria (Illumina). Successful rRNA depletion was confirmed using an Agilent RNA 6000 Pico Chip (Agilent Technologies). The TruSeq® Stranded mRNA LT Sample Prep Kit (Illumina) was used to produce cDNA libraries, which were sequenced using a MiSeq Desktop Sequencer (Illumina). Raw sequence data were deposited under accession number PRJEB27566.

### Differential gene expression analysis

Sequencing reads were filtered using Trimmomatic‐0.36 and reads that did not pass the filter were discarded (Bolger *et al.*, 2014). Filtered reads were aligned using Burrows‐Wheeler aligner to the EAEC 042 chromosome (FN554766.1) and the EAEC 042 pAA2 plasmid (FN554767.1) (Chaudhuri *et al.*, 2010; Li and Durbin, 2010). The alignment of reads to genes was counted using featureCounts (Liao *et al.*, 2014). DESeq2 was used to determine differentially expressed genes (Love *et al.*, 2014). Genes were termed differentially expressed if there was a >1 log_2_‐fold difference and an adjusted *p*‐value < 1E‐5 between wild‐type EAEC 042 and the Δ*aggR *mutant.

### qRT‐PCR

For qRT‐PCR analysis, overnight cultures of EAEC 042 pBAD24, EAEC 042 Δ*aggR* pBAD24 and EAEC 042 Δ*aggR* pBAD/*aggR*, in triplicate, were used to inoculate 4 ml DMEM high glucose supplemented with 100 μg ml^–1^ carbenicillin to a final OD_600_ of 0.05. Cultures were grown at 37°C with shaking as described above. At an OD_600_ of 0.4, L‐arabinose was added to a final concentration of 2%. Cultures were grown for 1 hour and RNA was extracted as described above. DNA was removed using TURBO DNA‐free™ (Ambion). RNA was reverse transcribed to cDNA using the Tetro cDNA Synthesis Kit (Bioline). Reactions for qRT‐PCR were prepared using the manufacturer’s instructions for the Brilliant III Ultra‐Fast SYBR® Green QPCR Master Mix (Agilent Technologies) and primers are detailed in Table S7. Relative gene expression was calculated using the 2^–ΔΔCT ^method (Livak and Schmittgen, 2001), with the *polA* gene used as a reference.

### Motility assays

Triplicate cultures of EAEC 042 Δ*aggR *pBAD24 and EAEC 042 Δ*aggR *pBAD/*aggR* were grown from overnight cultures to an OD_600_ of 1, each culture was inoculated into the centre of LB 0.25% agar plate supplemented with 0.2% L‐arabinose and incubated for 16 hours at 37°C. Plates were assessed for a difference in motility.

### Promoter fragment and plasmid construction

The promoter fragments *aafD*100, *afaB*100, *aggD*100, *aap*100, *aatP*100 and *aaiA*100 were amplified by PCR using the primer pairs listed in Table S7 with EAEC 042 or EAEC 17‐2 genomic DNA as template. The *aggD*101*, agg3D*100 and *agg4D*100 promoter fragments from EAEC strains C227‐11, 55989 and C1010‐00, respectively, were synthesized by Invitrogen Life Technologies. All DNA fragments are flanked by EcoRI and HindIII sites to facilitate cloning into pRW50 to generate *lacZ *transcriptional fusions. For shorter fragments, amplification was carried out using pRW50/*aafD*100, pRW50/*afaB*100 and pRW50/*aggD*100 as a template with the respective primers detailed in Table S7. Point mutations were introduced into fragments using megaprimer PCR, when necessary (Sarkar and Sommer, 1990). All constructs were verified by Sanger DNA sequencing.

### Bioinformatic analysis of DNA sequences

DNA target sites for the binding of AggR and the closely related Rns protein have been previously investigated, using *in vivo* and *in vitro* approaches (Morin *et al.*, 2010; Munson, 2013). From these studies, it has been proposed that the potential AggR‐binding site consensus sequence is 5′‐AnnnnnnTATC‐3′. Thus, based on this consensus, promoter fragments were screened for potential AggR‐binding sites on both strands, allowing for one mismatch to this consensus sequence. When predicted sites were found not to be necessary for AggR‐mediated regulation, as judged by deletion analysis, they were discounted. Potential AggR‐binding sequences, present in the smallest AggR‐regulated fragment, were then investigated using mutational analysis to identify the functional site.

The WebLogo motifs for the AggR‐binding site consensus sequence and AggR‐dependent promoter organization were generated by the WebLogo server (http://weblogo.berkeley.edu/logo.cgi) (Crooks *et al.*, 2004) using sequences from the EAEC 042 *aafD*,* afaB*,* aap*,* aatP, aaiA *and* aggR *promoters, the EAEC 17‐2 *aggD* promoter, the EAEC 55989 *agg3D* promoter and the EAEC C1010‐00 *agg4D* promoter (Fig. 5) (Morin *et al.*, 2010; Yasir, 2017). The 3D models of DNA promoter architecture, for the EAEC 17‐2 *aggD *and EAEC 042 *aafD *and *afaB *promoters, were produced by the model.it server using standard parameters (http://pongor.itk.ppke.hu/dna/model_it.html#/modelit_intro) (Munteanu *et al.*, 1998) and PyMOL (Schrodinger, 2010).

To identify AggR‐dependent promoters from our RNA‐seq data (Tables S2 and S3) 600 bp of DNA upstream of the first gene in each operon was searched for AggR‐binding sites, using the consensus sequence WWWWWWWTATC (Fig. 7A), only allowing two mismatches in the A/T rich tract and no mismatches in the conserved TATC motif. The presence of a −10 element was then examined by determining if there was a suitable match to the −10 region consensus sequence (TGnTATAAT) at a spacing of 21 to 23 bp, ensuring that first A in the −10 hexamer was present, as this is an important determinant of promoter strength (Browning and Busby, 2004).

### Assays of promoter activity

To assay the expression from promoter derivatives cloned into the *lac *expression vector pRW50, *E. coli* K‐12 BW25113 Δ*lac* strain was transformed with each construct and β‐galactosidase activity was measured as described in our previous work (Jayaraman *et al.*, 1987). AggR was expressed from pBAD/*aggR*, which carries *aggR *cloned downstream of the arabinose inducible promoter, *paraBAD* (Sheikh *et al.*, 2002). Cells were grown in LB medium at 37°C with shaking to mid‐logarithmic phase (OD_650 _= 0.4–0.6) and 0.2% w/v arabinose was included in the medium to induce AggR expression, where appropriate. β‐galactosidase activities are expressed as nmol of ONPG hydrolysed min^–1^ mg^–1^ dry cell mass and each activity is the average of three independent determinations.

### Primer extension assay

Primer extension analysis was carried our as described in our previous work (Lloyd *et al.*, 2008). *E. coli* K‐12 BW25113 cells, carrying various pRW50 derivatives and either pBAD/*aggR *or pBAD24, were grown in LB medium, containing 0.2% w/v arabinose, until mid‐logarithmic phase. RNA was extracted using an RNeasy Kit (Qiagen) and hybridized to ^32^P end‐labelled D49724 primer, which corresponds to sequence downstream of the HindIII site in pRW50 (Table S7). Primer extension products were run on a 6% denaturing polyacrylamide gel, containing 1 × TBE, and were analysed using a Bio‐Rad Molecular Imager FX and Quantity One software (Bio‐Rad). Gels were calibrated using an M13 sequence ladder, which was generated using a T7 sequencing kit (USB) with single‐stranded M13mp18 phage DNA and the M13 Universal Primer (Table S7).

## Conflict of Interest

The authors declare no conflict of interest.

## Author Contributions

DFB, SJWB, MP, IRH and MY conceived and designed the research project. MY, CI, RA and REG performed the experiments, aided by DFB, JRH and PS. MY, CI, SJWB, IRH and DFB wrote the manuscript with input from all authors.
