# Supplementary material for: Organization and architecture of AggR‐dependent promoters from enteroaggregative Escherichia coli
Source: Mol Microbiol. 2018 Dec 18;111(2):534–51. doi: 10.1111/mmi.14172 (PMC6392122; doi:10.1111/mmi.14172)
Supplement: Supplementary file 1 [file MMI-111-534-s001.docx]

***For Molecular Microbiology* Edited by DFB on 14/11/2018**

**Organization and architecture of AggR-dependent promoters from Enteroaggregative *Escherichia coli***

**Supplementary Material**

Muhammad Yasir ^1,4^, Christopher Icke ^1^, Radwa Abdelwahab ^1, 2^, James R. Haycocks ^1^, Rita E. Godfrey ^1^, Pavelas Sazinas ^3^, Mark J. Pallen ^4^, Ian R. Henderson ^1^,

Stephen J. W. Busby ^1^* and Douglas F. Browning ^1^*

^1^ Institute of Microbiology and Infection, School of Biosciences, University of Birmingham, Birmingham, B15 2TT, UK.

^2^ Faculty of Medicine, Assiut University, Egypt.

^3^ Department of Biotechnology and Biomedicine, Technical University of Denmark, DK-2800 Kgs Lyngby, Denmark.

^4^ Quadram Institute Bioscience, Norwich Research Park, Norwich, NR4 7UA, UK.

* For correspondence: SJWB Email: [s.j.w.busby@bham.ac.uk](mailto:s.j.w.busby@bham.ac.uk) Tel: +44 (0)121-414-5439.

DFB: Email: [D.F.Browning@bham.ac.uk](mailto:D.F.Browning@bham.ac.uk) Tel: +44 (0)121-414-5435.

**Table S1. Strains, plasmids and promoter fragments used in this work.**

**Bacterial strains. Relevant genotype or description. Reference or source.**

BW25113 *E. coli* K-12 *lacI*^q^, *rrnBT14,* *∆lacZ*_WJ16_ ([Baba *et al.*, 2006](#_ENREF_1))

*hsdR514,∆araBAD*_AH33,_ *∆rhaBAD*_LD78_.

JCB387 *E. coli* K-12 Δ*nir* Δ*lac*  ([Page *et al.*, 1990](#_ENREF_9))

JCB38707 *E. coli* K-12 JCB387 *hns::kn* ([Browning *et al.*, 2000](#_ENREF_2))

EAEC 042 Wild type EAEC strain. Sm^R^, Tet^R^ and Cm^R^. ([Nataro *et al.*, 1995](#_ENREF_8))

Diarrhoeagenic, expresses AAF/II, EAST1, Pet,

Pic and harbours the pAA2 plasmid.

EAEC 042 ∆*aggR* EAEC strain 042 carrying a deletion in *aggR*. ([Sheikh et al., 2002](#_ENREF_11))

EAEC 17-2 Wild type EAEC strain. Non-diarrhoeagenic, ([Nataro *et al.*, 1995](#_ENREF_8))

expresses AAF/I and harbours the pAA plasmid.

**Bacterial plasmids.**

pRW50 A broad-host-range *lacZ* transcription fusion plasmid, ([Lodge *et al.*, 1992](#_ENREF_4))

which carries a tetracycline resistance cassette.

pBAD24 A pBAD vector derivative carrying suitable ([Guzman *et al.*, 1995](#_ENREF_3))

restriction sites to clone a gene of interest

under the control of the *araBAD* promoter. Carries

an ampicillin resistance cassette.

pBAD/*aggR* A pBAD derivative carrying *aggR* under the control ([Sheikh *et al.*, 2002](#_ENREF_11))

of the *araBAD* promoter.

**Promoters fragments. All fragments are flanked by EcoRI and HindIII sites.**

*aafD*100 A 433 bp DNA fragment from EAEC strain 042, This study

containing 13 bp of the *aafD* coding sequence

and 420 bp upstream.

*aafD*99 A derivative of *aafD*100 constructed by deleting This study

100 bp downstream of the EcoRI site.

*aafD*98 A derivative of *aafD*100 constructed by deleting This study

200 bp downstream of the EcoRI site.

*aafD*97 A derivative of *aafD*100 constructed by deleting This study

300 bp downstream of the EcoRI site.

*aafD*96 A derivative of *aafD*100 constructed by deleting This study

320 bp downstream of the EcoRI site.

*aafD*95 A derivative of *aafD*100 constructed by deleting This study

340 bp downstream of the EcoRI site.

*aafD*94 A derivative of *aafD*100 constructed by deleting This study

360 bp downstream of the EcoRI site.

*aafD*96-92*C*90*C* A derivative of *aafD*96 carrying the 92*C* and 90*C* This study

point mutations.

*aafD*96-90*C* A derivative of *aafD*96 carrying the 90*C* mutation. This study

*aafD*96-65*C* A derivative of *aafD*96 carrying the 65*C* mutation. This study

*aafD*96-99*A* A derivative of *aafD*96 carrying the 99*A* mutation. This study

*aafD*96-99*C* A derivative of *aafD*96 carrying the 99*C* mutation. This study

*aafD*96-99*T* A derivative of *aafD*96 carrying the 99*T* mutation. This study

*aaiA*100 A 479 bp DNA fragment from EAEC strain 042, This study

containing 13 bp of the *aaiA* coding sequence and

463 bp upstream.

*aap*100 A 262 bp DNA fragment from EAEC strain 042, This study

containing 12 bp of the *aap* coding sequence and

250 bp upstream.

*aatP*100 A 454 bp DNA fragment from EAEC strain 042, This study

containing 13 bp of the *aatP* coding sequence

and 441 bp upstream.

*afaB*100 A 413 bp DNA fragment from EAEC strain 042, This study

containing 13 bp of the *afaB* pseudogene and 400 bp

upstream.

*afaB*99 A derivative of *afaB*100 constructed by deleting This study

100 bp downstream of the EcoRI site.

*afaB*98 A derivative of *afaB*100 constructed by deleting This study

200 bp downstream of the EcoRI site.

*afaB*97 A derivative of *afaB*100 constructed by deleting This study

300 bp downstream of the EcoRI site.

*afaB*100-320*C*318*C* A derivative of *afaB*100 carrying the 320*C* and 318*C* This study

point mutations.

*afaB*100-293*C* A derivative of *afaB*100 carrying the 293*C* mutation. This study

*aafC*100 A 410 bp DNA fragment from EAEC strain 042, This study

containing 13 bp of the *aafC* coding sequence and

397 bp upstream.

*afaBC*100 A 1088 bp DNA fragment from EAEC strain 042, This study

containing 13 bp of the *aafC* coding sequence and

1075 bp upstream, which includes the *afaB* pseudogene

and its regulatory region.

*afaBC*99 A derivative of *afaBC*100 constructed by deleting This study

400 bp downstream of the EcoRI site.

*aggD*100 A 413 bp DNA fragment from EAEC strain 17-2, This study

containing 13 bp of the *aggD* coding sequence and

400 bp upstream.

*aggD*99 A derivative of *aggD*100 constructed by deleting This study

268 bp downstream of the EcoRI site.

*aggD*98 A derivative of *aggD*100 constructed by deleting This study

305 bp downstream of the EcoRI site.

*aggD*97 A derivative of *aggD*100 constructed by deleting This study

326 bp downstream of the EcoRI site.

*aggD*98-86*C*  A derivative of *aggD*98 carrying the 86*C* mutation. This study

*aggD*98-65*C*  A derivative of *aggD*98 carrying the 65*C* mutation. This study

*aggD*101 A 199 bp DNA fragment from EAEC strain C227-11, This study

containing 13 bp of the *aggD* coding sequence and

186 bp upstream.

*agg3D*100 A 540 bp DNA fragment from EAEC strain 55989, This study

carrying the *agg3D* promoter region.

*agg3D*100-307*C* A derivative of *agg3D*100 carrying the 307*C* mutation. This study

*agg3D*100-331*C*333*C* A derivative of *agg3D*100 carrying the 331*C* and 333*C* This study

substitutions.

*agg4D*100 A 497 bp DNA fragment from EAEC strain C1010-00, This study

carrying the *agg4D* promoter region.

*agg4D*100-186*C* A derivative of *agg4D*100 carrying the 186*C* mutation. This study

*agg4D*100-211*C*213*C* A derivative of *agg4D*100 carrying the 211*C* and 213*C* This study

substitutions.

*CCmelR* A derivative of the *melR* promoter containing a ([Webster *et al.*, 1988](#_ENREF_12))

consensus CRP site centred at position -41.5.

*DAM*20 A derivative of *CCmelR* constructed by transferring This study

the AggR-binding site from the *aafD* promoter. The

AggR-binding site is spaced 20 bp from the -10

element.

*DAM*21 A derivative of *CCmelR* constructed by transferring This study

the AggR-binding site from the *aafD* promoter. The

AggR-binding site is spaced 21 bp from the -10

element.

*DAM*22 A derivative of *CCmelR* constructed by transfering This study

the AggR-binding site from the *aafD* promoter. The

AggR-binding site is spaced 22 bp from the -10

element.

*DAM*23 A derivative of *CCmelR* constructed by transfering This study

the AggR-binding site from the *aafD* promoter. The

AggR-binding site is spaced 23 bp from the -10

element.

**Table S2: Plasmid pAA2 genes that are differentially expressed in the EAEC 042 Δ*aggR* mutant in comparison to EAEC 042 as determined by RNA-seq analysis.**

|  | | | | |
| --- | --- | --- | --- | --- |
| Gene Name ^a^ | Function | | log_2_ fold change | Adjusted p-value |
| EC042_pAA003* | hypothetical protein | | –4.54 | 1.51E–56 |
| EC042_pAA004* | putative isopentenyl-diphosphate delta-isomerase | | –4.13 | 4.81E–64 |
|  |  |  | |  |
| EC042_pAA005* | hypothetical protein | | –5.34 | 1.53E–103 |
| EC042_pAA005A* | hypothetical protein | | –5.56 | 2.78E–80 |
|  |  |  | |  |
| EC042_pAA006 | pseudogene - transposase | | –1.58 | 9.06E–10 |
|  |  |  | |  |
| *aatP** | permease | | –5.52 | 4.37E–50 |
| *aatA** | TolC-like protein | | –6.05 | 2.72E–85 |
| *aatB** | membrane fusion protein | | –4.68 | 2.68E–40 |
| *aatC** | ATPase | | –4.55 | 2.36E–48 |
| *aatD** | required for Aap secretion | | –3.47 | 2.06E–28 |
|  |  |  | |  |
| EC042_pAA019 | hypothetical protein pseudogene | | –3.60 | 8.04E–50 |
| EC042_pAA020* | hypothetical protein | | –3.28 | 5.15E–41 |
| *shf** | polysaccharide deacetylase | | –3.04 | 1.23E–32 |
| EC042_pAA022* | glycosyl transferase | | –3.24 | 1.42E–28 |
| *virK* | virulence protein | | –3.01 | 1.79E–29 |
|  |  |  | |  |
| *aafB** | afimbrial adhesin | | –4.53 | 5.18E–43 |
| *aafC** | aggregative adherence fimbria II usher protein | | –4.53 | 6.08E–65 |
| *afaB** | pseudogene | | –6.68 | 2.91E–144 |
|  |  |  | |  |
| EC042_pAA033 | transposase | | –2.28 | 2.62E–06 |
|  |  |  | |  |
| *aafD** | chaperone protein | | –7.49 | 8.21E–95 |
| EC042_pAA047* | hypothetical protein | | –9.19 | 3.65E–198 |
| *aafA** | major fimbrial subunit of aggregative adherence fimbria II | | –8.16 | 1.51E–208 |
|  |  |  | |  |
| EC042_pAA051 | transposase | | –4.94 | 1.37E–35 |
|  |  |  | |  |
| *aggR** | transcriptional activator | | –5.47 | 2.40E–111 |
|  |  |  | |  |
| EC042_pAA053 | pseudogene - transposase | | –1.65 | 1.28E–10 |
|  |  |  | |  |
| *aap** | dispersin | | –9.36 | 1.51E–208 |
|  |  |  | |  |
| *aar** | AggR antivirulence factor | | –3.55 | 6.46E–33 |
|  |  |  | |  |
| EC042_pAA061 | hypothetical protein | | –3.25 | 9.29E–24 |
|  |  |  | |  |
| EC042_pAA105 | pseudogene - transposase | | –2.05 | 5.12E–10 |

^a^ Genes in the same operon have been grouped together. Consecutive genes in the table that are not in the same operon are separated by a blank line.

* Genes reported to be differentially expressed by microarray analysis ([Morin *et al.*, 2013](#_ENREF_5)).

**Table S3: Chromosomal genes that are differentially expressed in the EAEC 042 Δ*aggR* mutant in comparison to EAEC 042 as determined by RNA-seq analysis.**

| Gene Name ^a^ | Function | log_2_ fold change | Adjusted p-value |
| --- | --- | --- | --- |
| *phoA* | alkaline phosphatase | 1.44 | 4.97E-09 |
|  |  |  |  |
| *flu1* | antigen 43 | 1.99 | 8.24E-20 |
|  |  |  |  |
| *flu2* | antigen 43 | 2.32 | 4.49E-14 |
|  |  |  |  |
| EC042_4803 | antigen 43 | 4.29 | 3.63E-81 |
|  |  |  |  |
| *bssS* | biofilm regulator | –2.76 | 2.29E-15 |
|  |  |  |  |
| *flgN* | flagellar biosynthesis protein | –2.12 | 4.21E-09 |
| *flgM* | anti-sigma factor | –1.97 | 4.60E-06 |
| *flgA* | flagellar basal body P-ring formation protein | –3.29 | 2.92E-22 |
|  |  |  |  |
| *flgB* | flagellar basal-body rod protein | –6.05 | 1.72E-51 |
| *flgC* | flagellar basal-body rod protein | –5.60 | 2.40E-47 |
| *flgD* | flagellar biosynthesis, initiation of hook assembly | –5.19 | 2.67E-42 |
| *flgE* | flagellar hook protein | –5.23 | 1.06E-52 |
| *flgF* | flagellar basal-body rod protein | –4.27 | 1.34E-31 |
| *flgG* | flagellar basal-body rod protein | –4.33 | 6.68E-40 |
| *flgH* | flagellar L-ring protein | –3.32 | 3.01E-21 |
| *flgI* | flagellar P-ring protein | –2.74 | 3.81E-20 |
| *flgJ* | putative peptidoglycan hydrolase | –2.78 | 6.54E-19 |
| *flgK* | peptidoglycan hydrolase | –2.37 | 1.80E-14 |
| *flgL* | flagellar hook-filament junction protein 1 | –2.17 | 9.47E-11 |
|  |  |  |  |
| *cspI* | cold shock-like protein | –2.02 | 4.47E-07 |
|  |  |  |  |
| *anmK* | anhydro-N-acetylmuramic acid kinase | –1.18 | 6.58E-06 |
|  |  |  |  |
| *flhA* | flagella biosynthesis protein | –2.13 | 1.40E-11 |
| *flhB* | flagellar biosynthesis protein | –3.13 | 4.47E-15 |
|  |  |  |  |
| *cheW* | chemotaxis protein | –1.86 | 6.29E-07 |
| *cheA* | chemotaxis protein | –2.03 | 8.37E-11 |
| *flhC* | flagellar biosynthesis protein | –2.13 | 1.34E-17 |
| *flhD* | flagellar transcriptional activator | –2.05 | 3.35E-18 |
|  |  |  |  |
| *fliZ* | DNA-binding transcriptional regulator | –3.35 | 6.62E-18 |
| *fliA* | flagellar sigma factor | –5.09 | 3.97E-38 |
|  |  |  |  |
| *fliE* | flagellar hook-basal body complex protein | –2.93 | 1.88E-17 |
|  |  |  |  |
| *fliF* | flagellar M-ring protein | –4.99 | 2.38E-47 |
| *fliG* | flagellar motor switch protein | –4.48 | 3.43E-36 |
| *fliH* | flagellar assembly protein | –4.12 | 2.30E-31 |
| *fliI* | flagellum-specific ATP synthase | –4.11 | 3.44E-28 |
| *fliJ* | rod/hook and filament chaperone | –3.62 | 8.58E-22 |
| *fliK* | flagellar hook-length control protein | –3.90 | 4.79E-22 |
| *fliL* | flagellar basal body-associated protein | –4.08 | 1.13E-28 |
| *fliM* | flagellar motor switch protein | –4.43 | 5.29E-42 |
| *fliN* | flagellar motor switch protein | –4.19 | 2.08E-24 |
| *fliO* | flagellar biosynthesis protein | –3.27 | 9.41E-14 |
| *fliP* | flagellar biosynthetic protein | –2.63 | 1.32E-08 |
|  |  |  |  |
| EC042_2219 | hypothetical protein | –2.07 | 1.07E-07 |
|  |  |  |  |
| EC042_2249 | hypothetical protein | –3.00 | 7.94E-12 |
|  |  |  |  |
| EC042_3179A | hypothetical protein | –1.57 | 6.27E-08 |
| EC042_3180 | hypothetical protein | –2.88 | 1.09E-30 |
| EC042_3181 | putative transcriptional regulator | –2.44 | 4.95E-15 |
| EC042_3182* | ParB-like nuclease | –3.38 | 4.19E-45 |
| EC042_3183 | hypothetical protein | –4.35 | 2.26E-45 |
| EC042_3184* | hypothetical protein | –3.73 | 2.43E-25 |
| EC042_3185 | Pseudogene | –3.99 | 6.55E-34 |
| EC042_3187 | putative helicase | –4.09 | 1.86E-78 |
| EC042_3188 | Transposase | –4.91 | 6.15E-61 |
|  |  |  |  |
| EC042_3476 | hypothetical protein | –2.27 | 5.13E-06 |
|  |  |  |  |
| EC042_4006* | putative exported protein | –4.16 | 9.37E-43 |
|  |  |  |  |
| EC042_4430 | putative signal transduction protein | –2.89 | 1.59E-23 |
|  |  |  |  |
| *aaiA*/ EC042_4562* | putative type VI secretion protein | –4.41 | 2.72E-74 |
| *aaiB*/ EC042_4563* | putative type VI secretion protein | –4.14 | 6.55E-59 |
| *aaiC*/ EC042_4564* | putative type VI secretion protein | –3.99 | 2.88E-43 |
| *aaiD*/ EC042_4565* | putative type VI secretion protein | –3.33 | 1.00E-34 |
| *aaiE*/ EC042_4566* | putative type VI secretion protein | –3.14 | 2.04E-29 |
| *aaiF*/ EC042_4568* | putative type VI secretion protein | –3.00 | 2.24E-27 |
| *aaiG*/ EC042_4569* | putative type VI secretion protein | –3.21 | 1.51E-33 |
| *aaiH*/ EC042_4570* | putative type VI secretion protein | –3.16 | 4.03E-41 |
| *aaiI*/ EC042_4571* | putative type VI secretion protein | –2.75 | 1.40E-20 |
| *aaiJ*/ EC042_4572* | putative type VI secretion protein | –2.86 | 3.34E-32 |
| *aaiK*/ EC042_4573* | putative type VI secretion protein | –2.99 | 1.05E-35 |
| *aaiL*/ EC042_4574* | putative type VI secretion protein | –2.90 | 1.96E-27 |
| EC042_4574A* | putative type VI secretion protein | –2.74 | 1.64E-28 |
| *aaiM*/ EC042_4575* | putative type VI secretion protein | –2.86 | 1.46E-33 |
| *aaiN*/ EC042_4576* | putative type VI secretion protein | –2.94 | 1.48E-26 |
| *aaiO*/ EC042_4577* | putative type VI secretion protein | –2.78 | 6.92E-27 |
| EC042_4578 | pseudogene - transposase | –1.22 | 8.93E-06 |
| EC042_4579A | pseudogene - transposase | –1.88 | 1.18E-12 |
| *aaiR*/ EC042_4580* | hypothetical protein | –1.21 | 1.39E-07 |
| *aaiS*/ EC042_4581* | hypothetical protein | –2.19 | 6.55E-18 |
| EC042_4581A | pseudogene - transposase | –1.98 | 2.46E-16 |
| EC042_4581B | pseudogene - transposase | –1.67 | 4.97E-09 |
| *aaiT*/ EC042_4582* | hypothetical protein | –1.91 | 1.45E-17 |
| *aaiU*/ EC042_4583* | hypothetical protein | –1.70 | 6.01E-16 |
| EC042_4584 | pseudogene - transposase | –1.67 | 1.86E-08 |
| EC042_4585 | transposase | –1.68 | 6.97E-09 |
| EC042_4585A | pseudogene - transposase | –1.83 | 1.26E-08 |
| EC042_4587 | pseudogene - transposase | –1.57 | 3.54E-09 |

^a^ Genes in the same operon have been grouped together. Consecutive genes in the table that are not in the same operon are separated by a blank line.

* Genes reported to be differentially expressed by microarray analysis ([Morin *et al.*, 2013](#_ENREF_5)).

**Table S4. Relative gene expression of genes in EAEC 042 pBAD, EAEC 042 Δ*aggR* pBAD, and EAEC 042 Δ*aggR* pBAD/ *aggR* as determined by qRT-PCR.**

| Relative gene expression | *aap* | *fliA* | *flgB* | *fliC* | EC042_4803 |
| --- | --- | --- | --- | --- | --- |
| EAEC 042 pBAD | 1.0 | 1.0 | 1.0 | 1.0 | 1.0 |
| EAEC 042 Δ*aggR* pBAD | 0.0041± 0.0022 | 1.2±0.11 | 1.1±0.22 | 1.1±0.31 | 1.7±0.47 |
| EAEC 042 Δ*aggR* pBAD/*aggR* | 4.5±2.77 | 1.5±0.18 | 1.4±0.03 | 1.5±0.26 | 1.4±0.25 |

**Table S5. Analysis of TCAAGT repeats in the *aggD* promoters from various *E. coli* strains.**

**Bacterial strain. No of repeats. Country of Isolation. Accession number.**

17-2 6 Chile U12894.1

2009EL-2050 12 Georgia CP003299.1

2009EL-2071 13 Georgia CP003302.1

FHI102 14 Norway LM995513.1

C227-11 15 Germany CP011332.1

**Table S6. Analysis of AggR-dependent expression at different growth temperatures.**

**β-galactosidase activity ^c^**

**Promoter fragment ^a^ Temperature ^b^ pBAD24 pBAD/*aggR* Fold Increase ^d^**

*aafD*100 37˚C 473 ± 5 10658 ± 588 22

*aafD*100 30˚C 456 ± 12 4563 ± 517 10

*afaB*100 37˚C 31 ± 0.4 516 ± 39 17

*afaB*100 30˚C 39 ± 0.8 300 ± 85 8

^a^ All promoter fragments were cloned into the *lacZ* expression vector pRW50.

^b^ BW25113 cells were grown in LB medium in presence of 0.2% w/v arabinose at either 37˚C or 30˚C.

^c^ β-galactosidase activities are expressed as nmol of ONPG hydrolysed min^-1^ mg^-1^ dry cell mass. Each activity is the average of three independent determinations and standard deviations are shown.

^d^ The fold increase in β-galactosidase activity due to the expression of AggR is given for each promoter at each growth temperature.

**Table S7. DNA Primers used in this work. Primers (all are shown 5' to 3').**

**Primer name. Sequence ^a^.**

*aap*-1 TGGAACGCAGATAATGTG

*aap*-2 CATTAAGGCCTTGCATAC

*filA*-1 ATATTGCCGATTATCGCC

*filA*-2 ACCAGTTCAATGCTATCG

*filC*-1 TGATGGTGAAACCATCAC

*filC*-2 AGTATTGGCTGTTTCACC

*flgB*-1 GACGATGACCTCAACGCAAC

*flgB*-2 ATTACCGTCAAGCGAAGGCT

*polA*-1 TGGCGATAAAGATATGGC

*polA*-2 GTACTTATTCACCACCTC

4803-1 ATCCACATTTGTGGGTAC

4803-2 ATGTGATTCCATACCAGC

D10520 CCCTGCGGTGCCCCTCAAG

D10527 GCAGGTCGTTGAACTGAGCCTGAAATTCAG

D49724 GGTTGGACGCCCGGCATAGTTTTTCAGCAGGTCGTTG

M13 Universal Primer GTAAAACGACGGCCAGT

*aafD*100 Up GGGGGAATTCTGGTGCTTCAGGTGTGTGACATGGG

*aafD*100 Down GGGGGGAAGCTTTCCGTATTTTCATTTTATACATTCTCC

*aafD*99 Up GGGGGGGAATTCATGTACCGCCAACAATGCGGG

*aafD*98 Up GGGGGGGAATTCCGTAGTATTGCCAACTGAATC

*aafD*97 Up GGGGGGGAATTCCACCTGATTTATTCAATAAAGTCTG

*aafD*96 Up GGGGGGAATTCGTCTGCACAGTGGTGTTTATTTATC

*aafD*95 Up GGGGGGAATTCTTATCTTTTTAGTAACTTTGTTTTAAG

*aafD*94 Up GGGGGGAATTCTTTTAAGTAGCATATTAACTTAATCG

*aafD*96-92*C*90*C* GGGGGGGAATTCGTCTGCACAGTGGTGTTTATTCACCTTTTTAGTAAC

*aafD*96-65*C* GGGGGGGAATTCGTCTGCACAGTGGTGTTTATTTATCTTTTTAGTAAC

TTTGTTTTAAGTCGCATATTAAC

*aafD*96-90N GGGGGGGAATTCGTCTGCACAGTGGTnTTTATTTATCTTTTTAGTAAC

*aaiA* Up GGGGGAATTCTCCTTTGTTTTATGGATAGTTTTCTGC

*aaiA* Down GGGGAAGCTTGTGTATTGCTCATGTTTCTATCCTAC

*aap* Up GGGGGAATTCGTATTGTTAAATTACAAATGGATGG

*aap* Down GGGGAAGCTTTAATTTTTTTCATATGACTTCTCTC

*aatP*100 Up GGGGGGGAATTCCTTTCCAGAACCATACGAATCGC

*aatP*100 Down GGGGGGAAGCTTGCAAAGTTGTCATGTTGAATG

*afaB*100 Up GGGGGGGAATTCGACATGATAACGAATTAAGCAAGG

*afaB*100 Down GGGGGGAAGCTTGGGAAAATACTCTGGAGTTGGC

*afaB*99 Up GGGGGGAATTCATGTGACATTCCTGCACTG

*afaB*98 Up GGGGGGAATTCCCTTTCACAGGTAATGCAG

*afaB*97 Up GGGGGGAATTCGATGTATTGGTAGGGGCCT

*afaB*100-320*C*318*C* GTGTTTTTATCACCATTATGTGACATTCCTGC

*afaB*100-293*C* GTGTTTTTATCACCATTATGTGACATTCCTGC

*aafC*100 Up GGGGGGGAATTCAGGCTGACGATAAATGGGAGG

*aafC*100 Down GGGGGGAAGCTTATGTACATGTCATCATCACTG

*afaBC*99 GGGGGAATTCAGAGTATTTTCCCTTCATCTG

*aggD*100 Up GGGGGGGAATTCTTCTGGTGCTTCAGGTGTGTGACATGG

*aggD*100 Down GGGGGGAAGCTTTTCGAATCTTCATCGCTATCACTTG

*aggD*99 Up GGGGGAATTCAAATGGGGGCGGAATCCTAGTGTTAG

*aggD*98 Up GGGGGGGAATTCATGTTAAATAATGCTATTTTTTTAGCG

*aggD*97 Up GGGGGGGAATTCTTAGCGTTATATGATTTGAGTC

*aggD*98-86*C* GGGGGGGAATTCATGTTAAATAATGCTATTTTTTCAGCGTTATATG

*aggD*98-60*C* GATTTGAGTCTTTATCTAATCAAGTTCAAG

*agg3D*100 Up GGGGGGGAATTCCCCCAGACGAGAGTGACGG

*agg3D*100 Down GGGGGGAAGCTTTAACGGTGGCGTTATGATAAAAGG

*agg3D*100-331*C*333*C* CAATGTATGATATTTTTTCACCTCACAATAAATTGG

*agg3D*100-307*C* GGCGCACTATCTACTCTATATTAGAAATG

*agg4D*100 Up GGGGGGGAATTCGATGTACAGCATTCGGCTGTATCC

*agg4D*100 Down GGGGGGAAGCTTGCGTGTAGCCCCTATATGTATAGAAAAAAATTTTG

*agg4D*100-211*C*213*C* AATATTTTTATTTATTCACCTTTTTTTTGGGCGCTATG

*agg4D*100-185*C* GGGCGCTATGTTTTTCTAATCTTGAAAGATTTC

*DAM*20 GTGTTTATTTATCTTTTATCCCCTCACTCCTG

*DAM*21 GATCAGGTAAATGGTGTTTATTTATCTTTTATCCCCCTCACTCCTG

*DAM*22 GATCAGGTAAATGGTGTTTATTTATCTTTTATCCCCCCTCACTCCTG

*DAM*23 GTGTTTATTTATCTTTTATCCCCCCCTCACTCCTG

**^a^** Restriction sites are underlined

**Supplementary Figure legends**

**Fig. S1. Relative gene expression levels of various genes identified by RNA-seq analysis.**

The figure shows the relative expression of various genes identified in the RNA-seq analysis in wild type EAEC 042 and its Δ*aggR* derivative. EAEC 042 pBAD, EAEC 042 Δ*aggR* pBAD, and EAEC 042 Δ*aggR* pBAD/*aggR* cells were grown to an OD_600_ of 0.4 in DMEM high glucose medium and AggR production was induced, where appropriate, by the addition of L-arabinose to 2% for 1 hour prior to RNA extraction. The relative gene expression of *fliA*, *flgB*, *fliC*, and EC042_4803 (an Antigen-43 homologue) was calculated using qRT-PCR with *polA* as the reference gene and *aap* expression was included as a positive control. The figure shows that *fliA*, *flgB*, *fliC*, and EC042_4803 are not regulated by AggR.

**Fig. S2. Motility assays.**

The figure shows motility assays for EAEC 042 Δ*aggR* carrying either pBAD or pBAD/*aggR*. Logarithmically growing cultures were used to inoculate 0.25% LB agar plates supplemented with 0.2% L-arabinose in biological triplicate (1 to 3 vertically) and technical duplicate (horizontally). Plates were incubated at 37ºC for 16 hours. Note that AggR expression is induced in cells carrying pBAD/*aggR* by the addition of L-arabinose. The figure shows that EAEC motility is not affected by AggR expression.

Fig. S3 Analysis of AggR-regulated promoters from EAEC strain 042.

The figure shows a series of experiments to examine the AggR-dependent regulation of candidate genes selected from the RNA-seq analysis. For each gene ~400 bp of upstream DNA was cloned into the *lacZ* expression vector pRW50. The fragments analysed were A. *aafD*100, B. *afaB*100, C. *aap*100, D. *aatP*100 and E. *aaiA*100. Each panel illustrates the β-galactosidase activities measured in the *∆lac E. coli* K-12 strain BW25113, carrying pRW50 with the relevant cloned promoter fragment. Cells also carried either pBAD/*aggR* (grey bars) or pBAD24 (black bars) and were grown in LB medium in the presence (+) or absence (-) of 0.2% w/v arabinose. β-galactosidase activities are expressed as nmol of ONPG hydrolysed min^-1^ mg^-1^ dry cell mass. Each activity is the average of three independent determinations and standard deviations are shown for all data points.

Fig. S4. Analysis of the EAEC 042 *afaB-aafCB* operon promoter.

A. Organisation of the fimbrial genes at the *afaB-aafCB* locus on the pAA2 plasmid. The upper arrows show the arrangement of the *afaB* pseudogene and *aafC* on pAA2. The boxed region is expanded in the lower part of the panel and illustrates the fragments used in this study. Sequence upstream of *afaB* and *aafC* are in grey*,* the *afaB* pseudogene is shown by a dotted grey bar and the small region of *aafC*, included on fragments, is in black. All fragments were cloned into pRW50, using EcoRI and HindIII restriction sites.

B. The panel illustrates the β-galactosidase activities measured in the *∆lac E. coli* K-12 strain BW25113, carrying the *lacZ* expression vector pRW50, containing either *aafC*100, *afaB*100, *afaBC*100 or *afaBC*99 promoter fragments. Cells also carry either pBAD/*aggR* (grey bars) or pBAD24 (black bars) and were grown in LB medium in the presence (+) or absence (-) of 0.2% w/v arabinose. β-galactosidase activities are expressed as nmol of ONPG hydrolysed min^-1^ mg^-1^ dry cell mass. Each activity is the average of three independent determinations and standard deviations are shown for all data points.

Fig. S5. Comparison of the *aggD* promoters from EAEC strains 17-2 and C227-11.

A. The panel shows the DNA sequences of the *aggD*99 and *aggD*101 promoter fragments from the AggR-binding site to the start site of translation of *aggD*. Fragment *aggD*99 carries the *aggD* promoter from EAEC strain 17-2, whilst *aggD*101, the *aggD* promoter from EAEC strain C227-11. The AggR-binding sites are shown by solid black arrows, the -10 hexamer elements are underlined and the translational start codon (ATG) is in bold and italic. The number of 5' -TCAAGT-3' repeats is indicated for each fragment.

B. The panel illustrates β-galactosidase activities measured in the *∆lac E. coli* K-12 strain BW25113, containing the *lacZ* expression vector pRW50 carrying the *aggD101 and aggD99* promoter derivatives. Cells also carry either pBAD/*aggR* (grey bars) or pBAD24 (black bars) and were grown in LB medium in presence (+) or absence (-) of 0.2% w/v arabinose. β-galactosidase activities are expressed as nmol of ONPG hydrolysed min^-1^ mg^-1^ dry cell mass. Each activity is the average of three independent determinations and standard deviations are shown for all data points.

**Fig. S6. The DNA sequence of the EAEC 55989 *agg3D*100 and EAEC C1010-00 *agg4D*100 promoter fragments.**

The figure shows the DNA sequences of the A. *agg3D*100 and B. *agg4D*100 promoter fragments from EAEC strains 55989 and C1010-00, respectively. Both sequences are flanked by upstream EcoRI and downstream HindIII sites and are numbered from the base adjacent to the HindIII site. The proposed ‑10 elements are underlined and the initiating ATG codons are in bold. Potential AggR-binding sites are indicated by horizontal arrows and each site is aligned with the AggR-binding consensus ([Morin *et al.*, 2010](#_ENREF_6)). The location of the 307*C* and 331*C*/ 333*C* substitutions and the 186*C* and 211*C*/ 213*C* substitutions, which disrupt the -10 element and the functional AggR-binding site in *agg3D*100 and *agg4D*100, respectively, is shown.

Fig. S7. Mutational analysis of the AggR-binding site at the EAEC 042 *aafD* promoter.

A. The panel shows the base sequence of the *aafD*96 promoter fragment. The functional AggR-binding site is indicated by a solid horizontal arrow, the -10 hexamer is underlined and the EcoRI and HindIII restriction sites are boxed. Vertical arrows indicate the locations of various point mutations introduced into *aafD*96 at positions 99 and 90.

B. The panel illustrates β-galactosidase activities measured in the *∆lac E. coli* K-12 strain BW25113*,* containing various *aafD*96 promoter derivatives cloned into pRW50. Cells also carry either pBAD/*aggR* (grey bars) or pBAD24 (black bars) and were grown in LB medium in the presence (+) or absence (-) of 0.2% w/v arabinose. β-galactosidase activities are expressed as nmol of ONPG hydrolysed min^-1^ mg^-1^ dry cell mass. Each activity is the average of three independent determinations and standard deviations are shown for all data points.

Fig. S8. Modelling of AggR-dependent promoter architecture.

The figure shows 3D models of promoter architecture for the EAEC 17-2 *aggD* and EAEC 042 *aafD* and *afaB* promoters, produced using the model.it prediction tool ([Munteanu *et al.*, 1998](#_ENREF_7)) and PyMOL ([Schrodinger, 2010](#_ENREF_10)). The AggR-binding site is coloured pink and -10 element is blue.

**Fig. S9. Alignment of AggR-dependent promoters from different *E. coli* strains.** The figure shows the alignment of the EAEC 042 AggR-dependent promoters, upstream of A. *aggR*, B. *aatP*, C. *aap* and D. *aaiA*, with corresponding DNA sequences from various EAEC strains (*i.e.* 2009EL-2050, 2009EL-2071, 55989, C227-11 and 17-2, where appropriate). The location of the AggR-binding site and the -10 element for each promoter is shown, aligned with the relevant consensus sequences.

Fig. S10. Expression analysis of *afaB* and *aafD* promoter constructs in JCB387 and its *hns* mutant derivative, JCB38707.

A. The panel illustrates β-galactosidase activities measured in the *∆lac E. coli* K-12 strains JCB387 and JCB38707 (*hns::kn*), containing the *lacZ* expression vector pRW50 carrying the *afaB*100 promoter fragment.

B. The panel illustrates β-galactosidase activities measured in JCB387 and JCB38707 (*hns::kn*), containing pRW50 carrying the *aafD*96 promoter fragment. For both panels A. and B. cells also carry either pBAD/*aggR* (grey bars) or pBAD24 (black bars) and were grown in LB medium in presence (+) or absence (-) of 0.2% w/v arabinose. β-galactosidase activities are expressed as nmol of ONPG hydrolysed min^-1^ mg^-1^ dry cell mass. Each activity is the average of three independent determinations and standard deviations are shown for all data points.

Supplementary References

Baba, T., Ara, T., Hasegawa, M., Takai, Y., Okumura, Y., Baba, M., Datsenko, K.A., Tomita, M., Wanner, B.L. and Mori, H. (2006) Construction of *Escherichia coli* K-12 in-frame, single-gene knockout mutants: the Keio collection. *Mol Syst Biol* **2**: 2006 0008.

Browning, D.F., Cole, J.A. and Busby, S.J. (2000) Suppression of FNR-dependent transcription activation at the *Escherichia coli nir* promoter by Fis, IHF and H-NS: modulation of transcription initiation by a complex nucleo-protein assembly. *Mol Microbiol* **37**: 1258-1269.

Guzman, L.M., Belin, D., Carson, M.J. and Beckwith, J. (1995) Tight regulation, modulation, and high-level expression by vectors containing the arabinose PBAD promoter. *J Bacteriol* **177**: 4121-4130.

Lodge, J., Fear, J., Busby, S., Gunasekaran, P. and Kamini, N.R. (1992) Broad host range plasmids carrying the *Escherichia coli* lactose and galactose operons. *FEMS Microbiol Lett* **74**: 271-276.

Morin, N., Santiago, A.E., Ernst, R.K., Guillot, S.J. and Nataro, J.P. (2013) Characterization of the AggR Regulon in Enteroaggregative *Escherichia coli*. *Infect Immun* **81**: 122-132.

Morin, N., Tirling, C., Ivison, S.M., Kaur, A.P., Nataro, J.P. and Steiner, T.S. (2010) Autoactivation of the AggR regulator of enteroaggregative *Escherichia coli in vitro* and *in vivo*. *FEMS Immunol Med Microbiol* **58**: 344-355.

Munteanu, M.G., Vlahovicek, K., Parthasarathy, S., Simon, I. and Pongor, S. (1998) Rod models of DNA: sequence-dependent anisotropic elastic modelling of local bending phenomena. *Trends Biochem Sci* **23**: 341-347.

Nataro, J.P., Deng, Y., Cookson, S., Cravioto, A., Savarino, S.J., Guers, L.D., Levine, M.M. and Tacket, C.O. (1995) Heterogeneity of enteroaggregative *Escherichia coli* virulence demonstrated in volunteers. *J Infect Dis* **171**: 465-468.

Page, L., Griffiths, L. and Cole, J.A. (1990) Different physiological roles of two independent pathways for nitrite reduction to ammonia by enteric bacteria. *Arch Microbiol* **154**: 349-354.

Schrodinger, LLC, (2010) *The PyMOL Molecular Graphics System, Version 1.3r1*.

Sheikh, J., Czeczulin, J.R., Harrington, S., Hicks, S., Henderson, I.R., Le Bouguenec, C., Gounon, P., Phillips, A. and Nataro, J.P. (2002) A novel dispersin protein in enteroaggregative *Escherichia coli*. *J Clin Invest* **110**: 1329-1337.

Webster, C., Gaston, K. and Busby, S. (1988) Transcription from the *Escherichia coli melR* promoter is dependent on the cyclic AMP receptor protein. *Gene* **68**: 297-305.

**Fig. S1.**


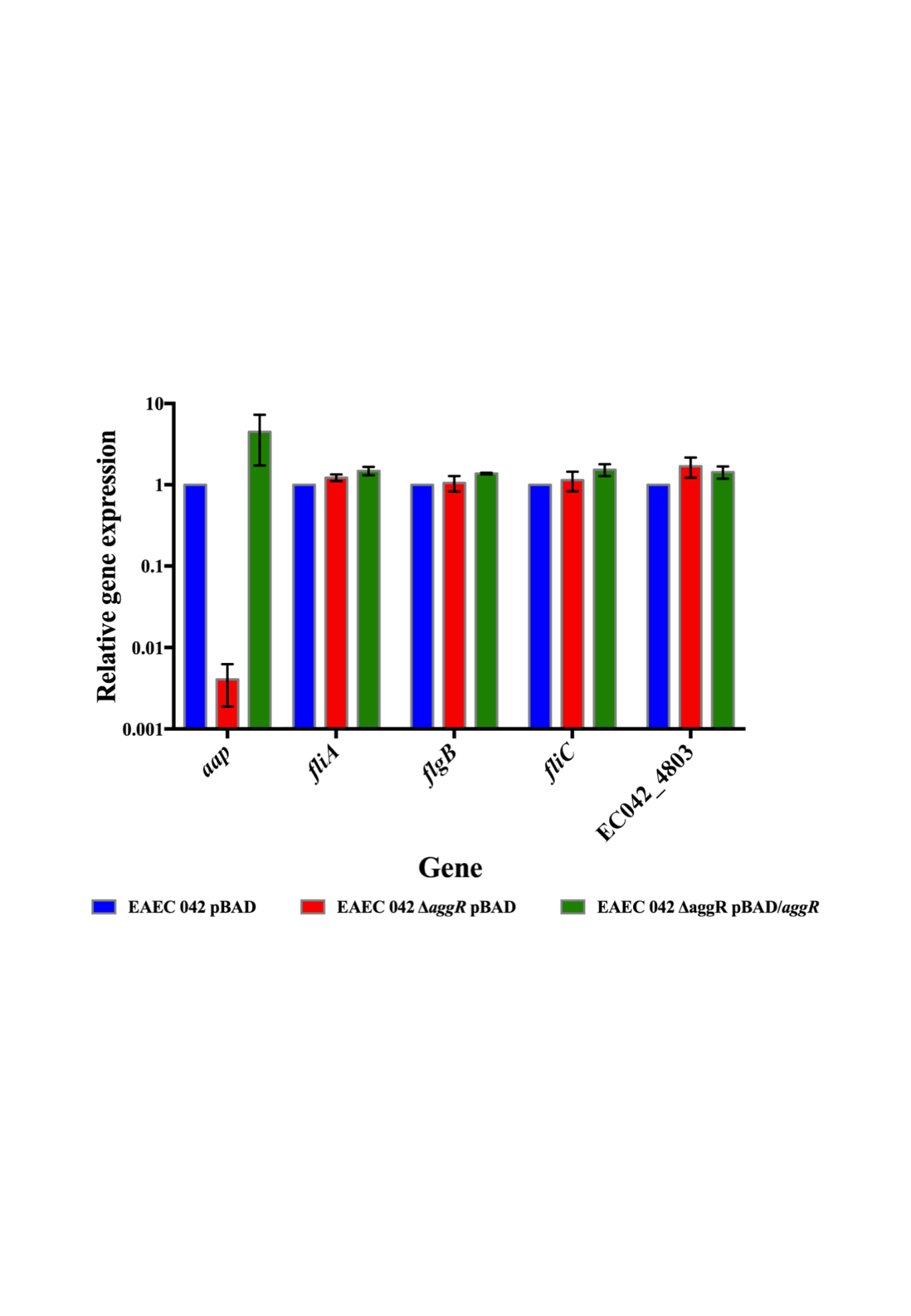


Fig. S2.


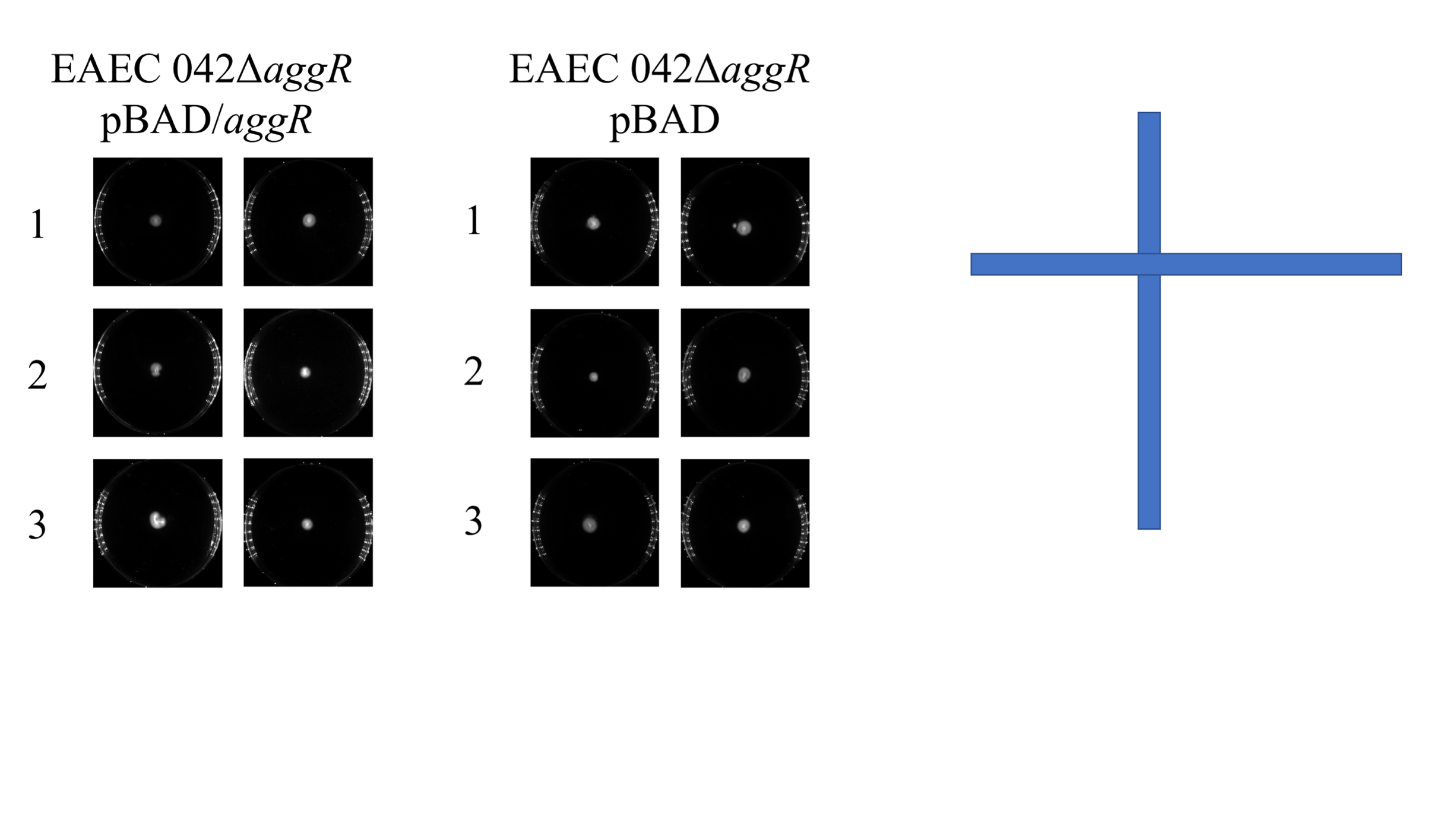


Fig. S3.


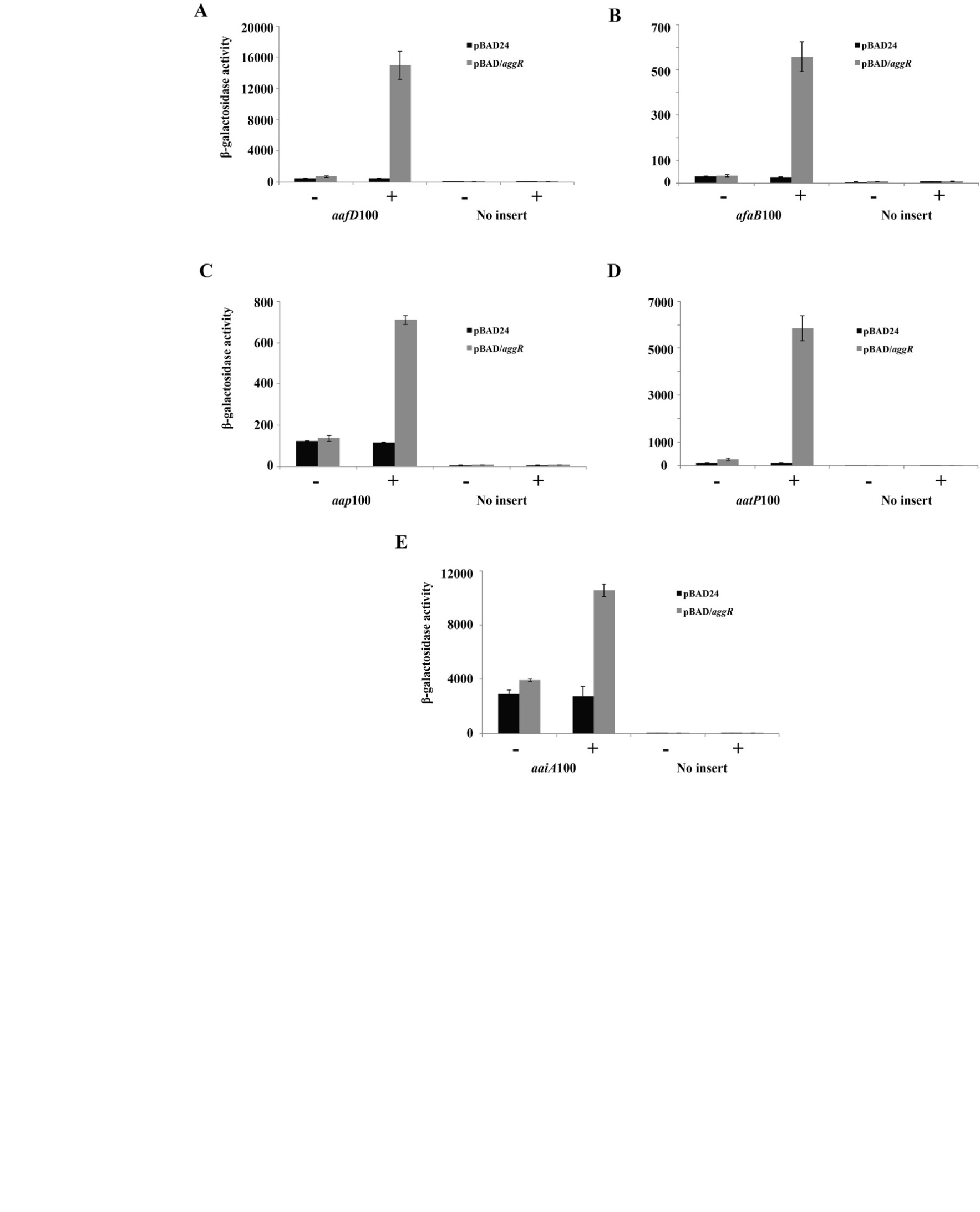


**Fig. S4.**


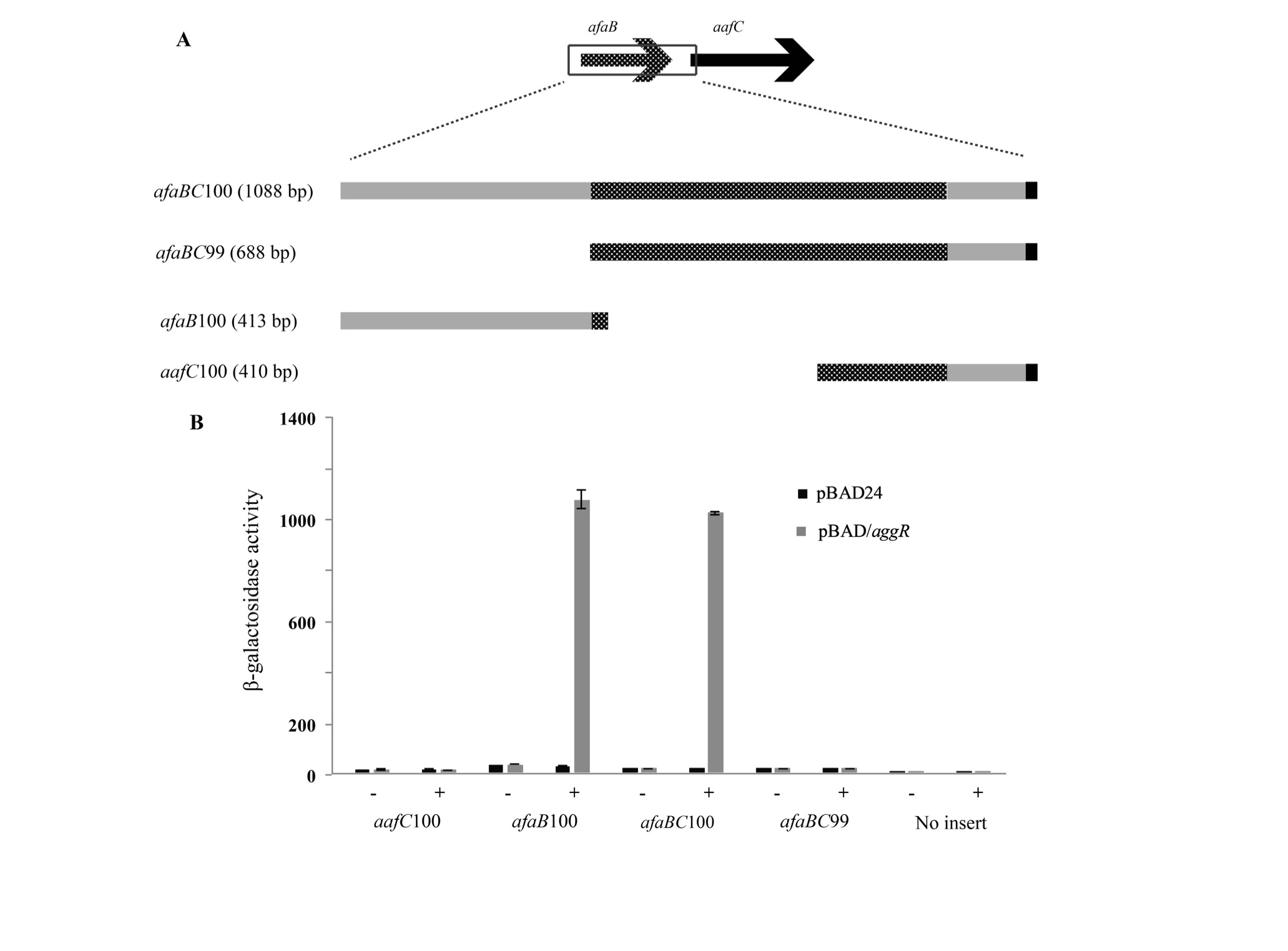


**Fig. S5.**

**
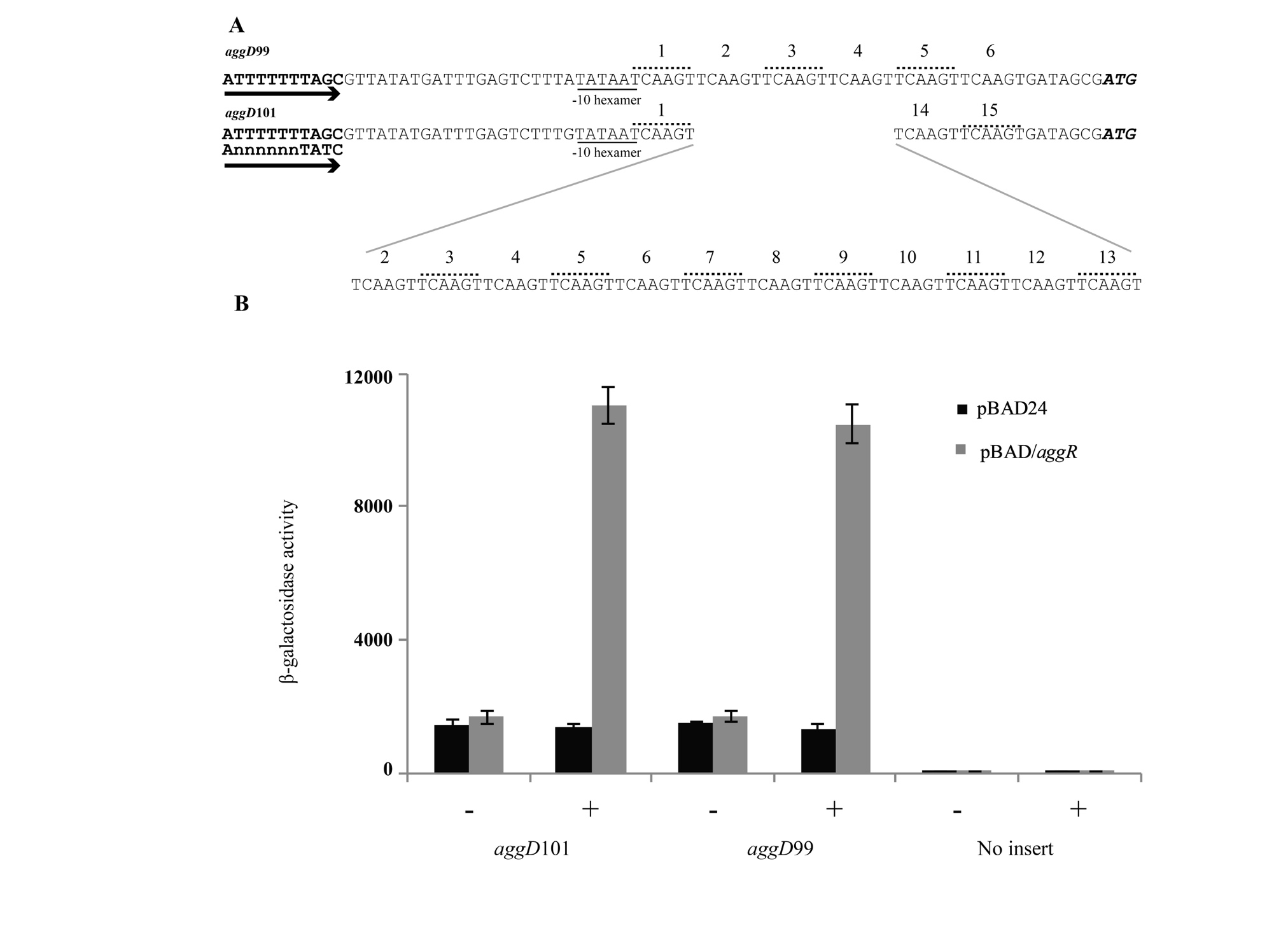
**

Fig. S6.

**
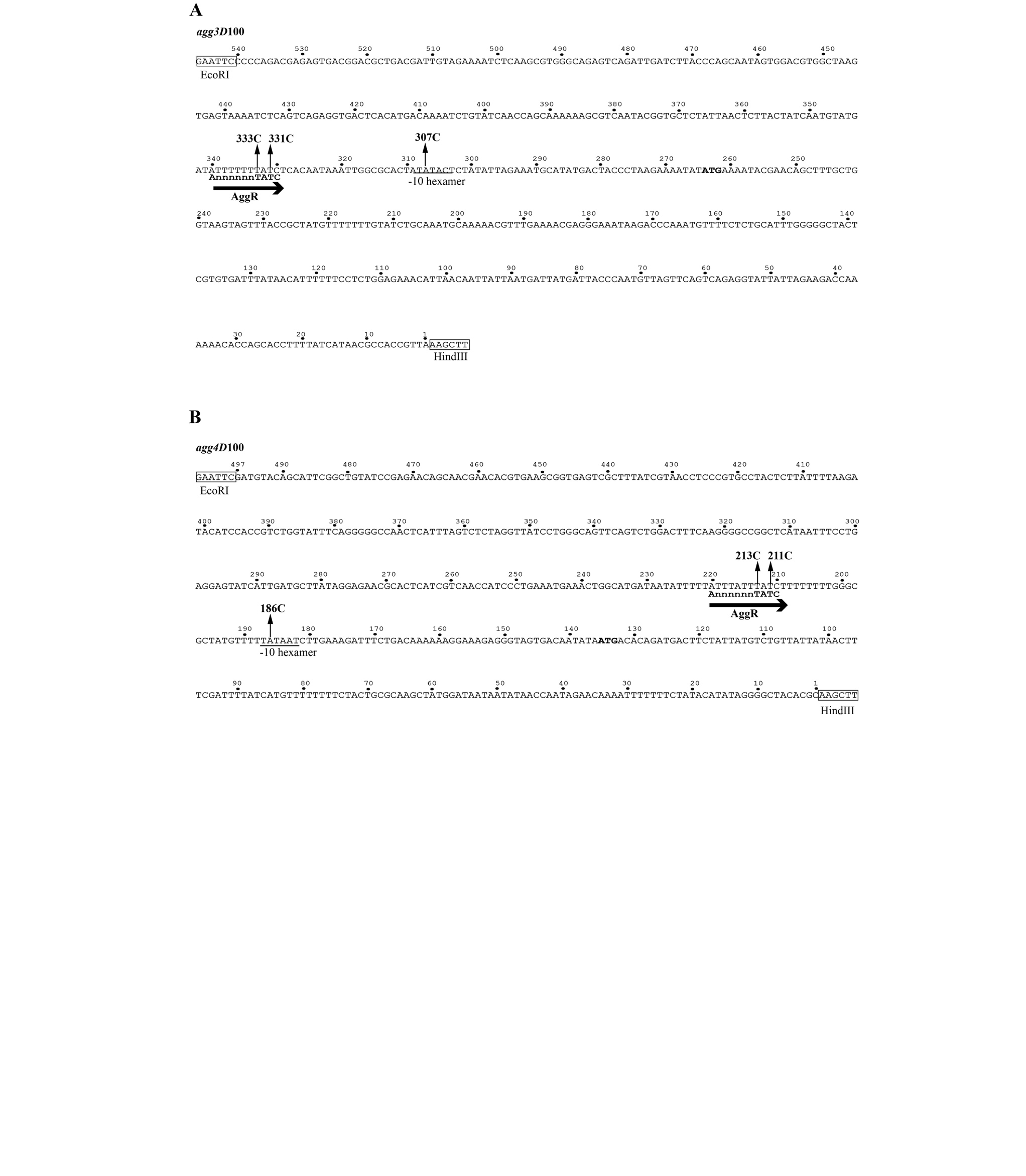
**

**Fig. S7.**


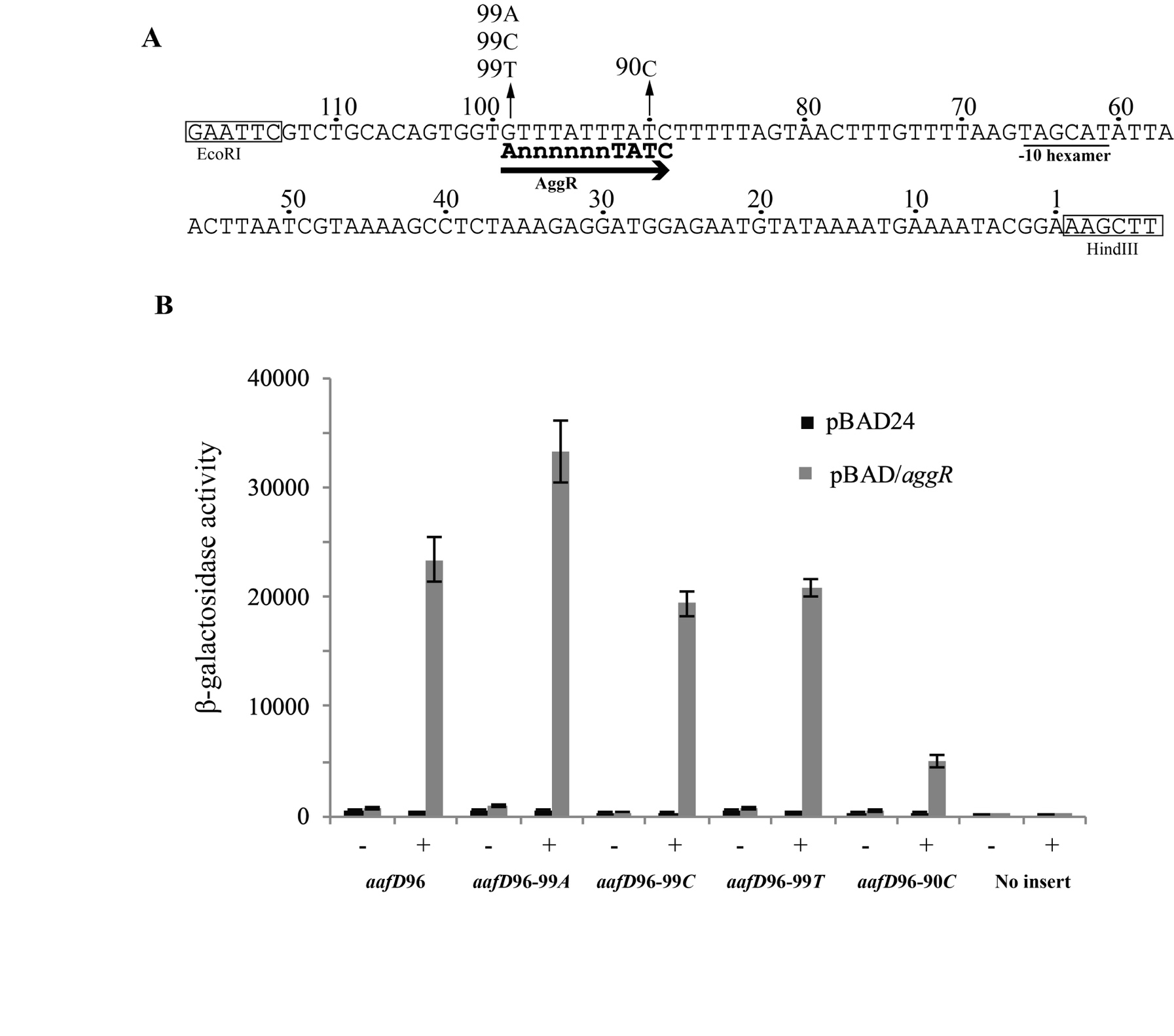


**Fig. S8.**


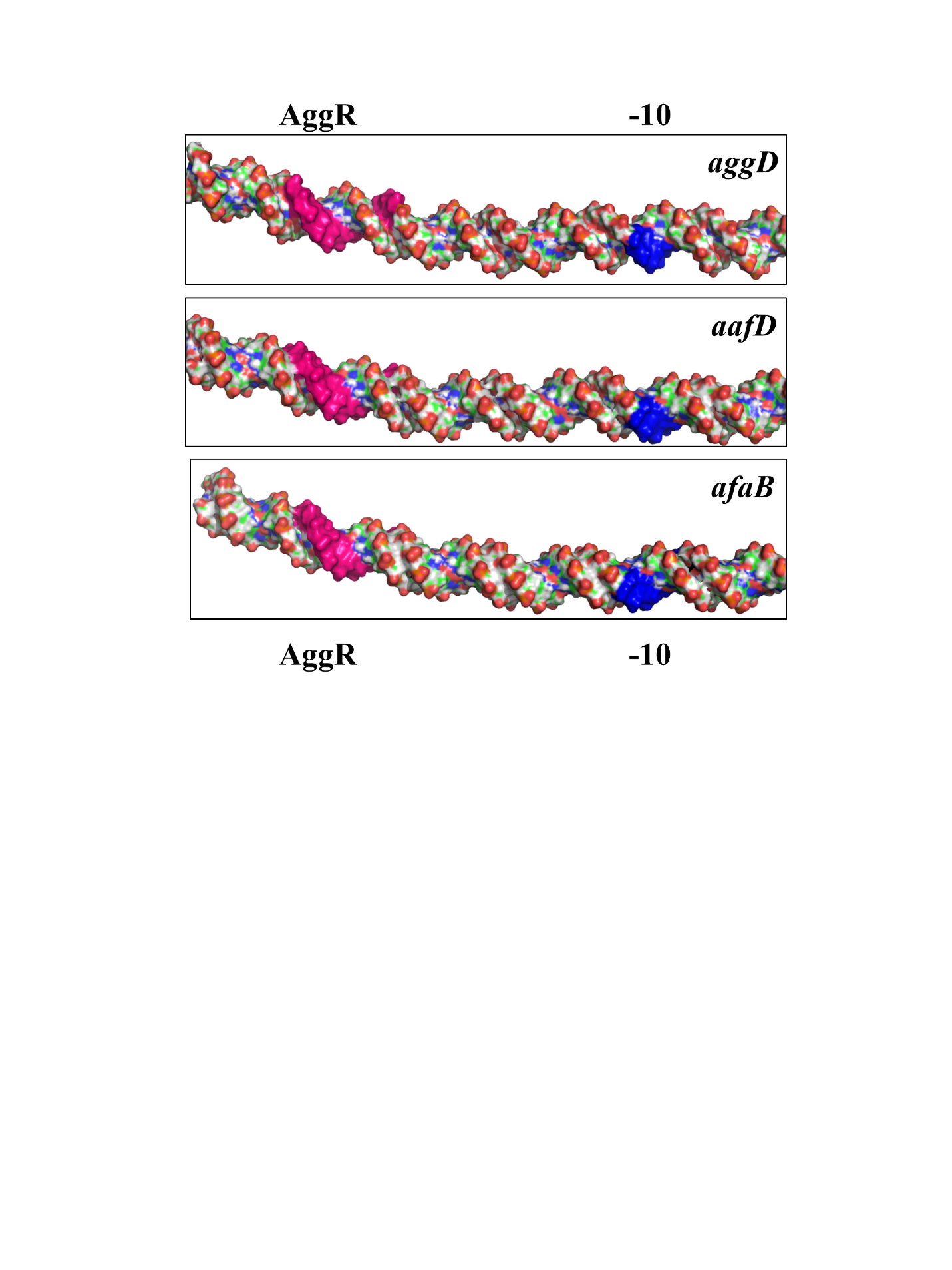


**Fig. S9.**

**A** *aggR*

**AggR 22bp -10**

2009EL-2071 CATATGAGTT**AAAAATTTATC**TTTTTATTGATAAGAGTTAGGTC**A**CTC**T**AACGCAGAGTT

2009EL-2050 CATATGAGTT**AAAAATTTATC**TTTTTATTGATAAGAGTTAGGTC**A**CTC**T**AACGCAGAGTT

C227-11 CATATGAGTT**AAAAATTTATC**TTTTTATTGATAAGAGTTAGGTC**A**CTC**T**AACGCAGAGTT

55989 TATATGAGTT**AAAAATTTATC**TTTTTATTGATAAGAGTTAGGTC**A**CTC**T**AACGCAGAGTT

17-2 TATATGAGTT**AAAAATTTATC**TTTTTATTGATAAGAGTTAGGTC**A**CTC**T**AACGCAGAGTT

042 TATATGAGTT**AAAAATATATC**TTTTTATTGATAAGAGTTAGGTC**AT**TC**T**AACGCAGA-TT

*************** **************************** *********** **

**WWWWWWWTATC TATAAT**

**B** *aatP*

**AggR 21bp -10**

C227-11 ATAAACTTAT**AATTATATATC**CCTTAGTTATTAATAGTCAGG**TA**C**A**T**T**ATATATAATGTT

2009EL-2071 ATAAACTTAT**AATTATATATC**CCTTAGTTATTAATAGTCAGG**TA**C**A**T**T**ATATATAATGTT

2009EL-2050 ATAAACTTAT**AATTATATATC**CCTTAGTTATTAATAGTCAGG**TA**C**A**T**T**ATATATAATGTT

55989 ATAAACTTAT**AATTATATATC**CCTTAGTTATTAATAGTCAGG**TA**C**A**T**T**ATATATAATGTT

042 ATAAACTTAT**A**G**TTATATATC**CCTTAGTTATTAATAGTTGGG**TA**C**A**T**T**ATATA--GTGTT

*********** ************************** ************* ****

**WWWWWWWTATC TATAAT**

**C** *aap*

**AggR 22bp -10**

55989 TATATGTTGC**TATTTTTTATC**TGACCGCAACTCTTTATTA**TG**C**TA**ACC**T**CCTAAAAGGAG

C227-11 TATATGTTGC**TATTTTTTATC**TGACCGCAACTCTTTATTA**TG**C**TA**ACC**T**CCTAAAAGGAG

2009EL-2050 TATATGTTGC**TATTTTTTATC**TGACCGCAACTCTTTATTA**TG**C**TA**ACC**T**CCTAAAAGGAG

2009EL-2071 TATATGTTGC**TATTTTTTATC**TGACCGCAACTCTTTATTA**TG**C**TA**ACC**T**CCTAAAAGGAG

042 TATATGTTGC**TATTTTTTATC**TGGCCGCAACTCTTATTTA**TG**C**TA**GCC**T**TCTAAAAGGAG

*********************** *********** ******** *** **********

**WWWWWWWTATC TGnTATAAT**

**D** *aaiA*

**AggR 23bp -10**

C227-11 ATTAATCAGC**AAAAAT**G**TATC**ACATGCTCACTTTCTTTTTA**TG**G**TAT**C**A**CTATATAGAAT

2009EL-2071 ATTAATCAGC**AAAAAT**G**TATC**ACATGCTCACTTTCTTTTTA**TG**G**TAT**C**A**CTATATAGAAT

2009EL-2050 ATTAATCAGC**AAAAAT**G**TATC**ACATGCTCACTTTCTTTTTA**TG**G**TAT**C**A**CTATATAGAAT

55989 ATTAATCAGC**AAAAAT**G**TATC**ACATGCTCACTTTCTTTTTA**TG**G**TAT**C**A**CTATATAGAAT

042 ATTAATCAGC**AAAAAT**G**TATC**ACATGCTCACTTTCTTTTTA**TG**G**TAT**C**A**CTATATAGAAT

************************************************************

**WWWWWWWTATC TGnTATAAT**

**Fig. S10**

**
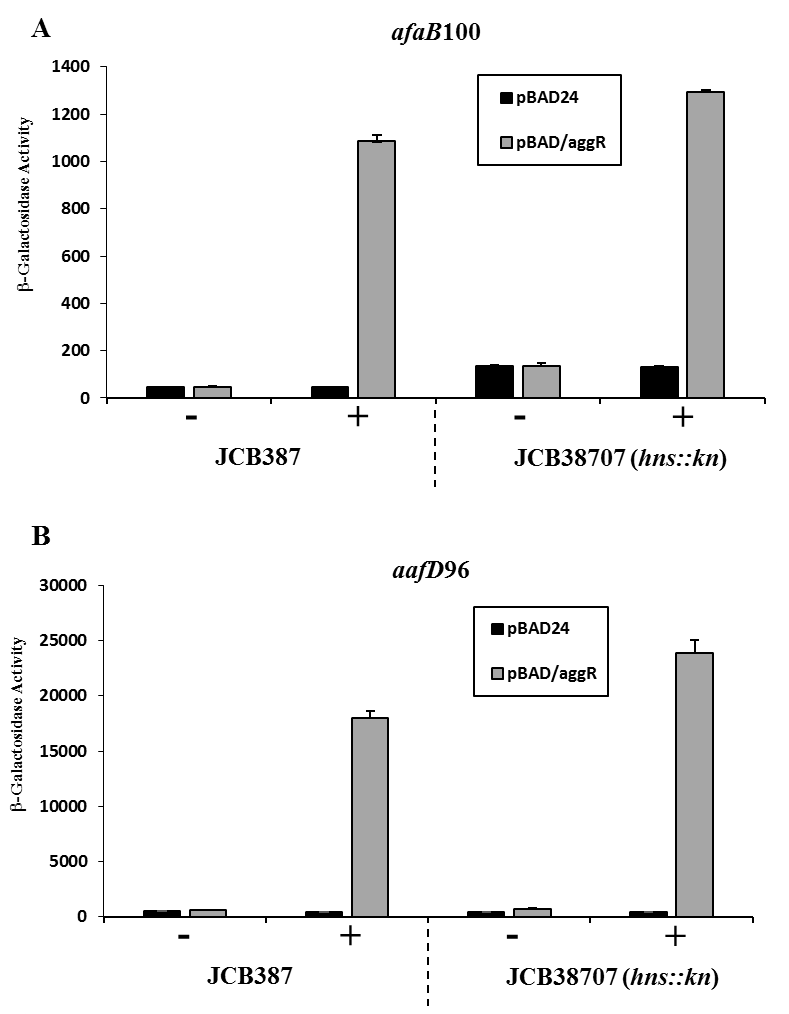
**
